# Assessing urban community parks from an age-friendly perspective: a multi-criteria decision-making approach

**DOI:** 10.3389/fpubh.2025.1663359

**Published:** 2025-11-07

**Authors:** Weidan Dong, Shuai Sun, Yinghang Fu

**Affiliations:** 1 Department of Architectural Engineering, Dongshin University, Naju, Republic of Korea; 2 School of Energy and Constructional Engineering, Shandong Huayu University of Technology, Dezhou, Shandong, China

**Keywords:** age-friendly, community park, evaluation system, AHP, entropy weight method, TOPSIS

## Abstract

**Introduction:**

With the acceleration of population ageing, community parks have become vital spaces for older adults’ daily activities. The age-friendliness of their spatial environment directly impacts the well-being of the older adult population. Scientifically evaluating the age-friendliness of community parks and proposing optimization strategies is crucial to promoting the physical and mental well-being of the older adults and enhancing the quality of urban public services.

**Methods:**

This study established an evaluation index system for the age-friendliness of community parks through field surveys, literature review, expert screening, and statistical validation. User perception data were collected via questionnaires, and a combination of the Analytic Hierarchy Process (AHP) and Entropy Weight Method was used to determine weights from both subjective and objective perspectives. Subsequently, the TOPSIS model was employed to conduct a comprehensive assessment of three typical community parks in Beijing.

**Results:**

The results indicate that path accessibility, ageing of leisure facilities, social space design, cultural, educational and recreational functions, and physical environment security are the most influential factors affecting the age-friendliness of community parks, with the highest weights. In contrast, intelligent service facilities, seasonal adaptability services, and community activities organization are relatively less prioritized by older adults. The findings reveal that the core needs of older adults in community park usage are centered on four key aspects: accessibility, recreation, social interaction, and safety. Furthermore, a comparative analysis revealed significant differences in overall age-friendliness across the three case parks, with Wanshou Park performing the best, Nanguan Park ranking second, and Shuangxiu Park ranking lowest.

**Discussion:**

Based on these findings, strategies to enhance the age-friendliness of community parks were proposed. This study provides both theoretical support and practical reference for the scientific assessment and optimization of community park age-friendliness, offering important insights for the adaptation of urban public spaces to an aging society.

## 1 Introduction

The global ageing process is accelerating, with the proportion of the older adult population expected to exceed 14% by 2038, entering a stage of deep ageing and exceeding 20% by 2070, entering a stage of super-ageing (1). China’s ageing population is characterized by a large-scale older adult population, a significantly accelerated ageing process, and continuously improving quality of the older adult population. According to data from the seventh national census conducted by the National Bureau of Statistics of China, the proportion of the population aged 65 and above reached 13.50%, with the population ageing already exceeding the global average. Compared with the sixth national census conducted in 2010, the population aged 60 and above increased by 5.44%, and the proportion of those aged 65 and above increased by 4.63% (2). The World Health Organization (WHO) has defined “healthy aging,” emphasizing the importance of environmental support (3). The “Decade of Healthy Aging Action Plan (2020–2030)” establishes a global action framework, requiring countries to promote the functional capabilities of older people by optimizing physical and social environments (4). China has actively responded and implemented measures to promote the development of age-friendly cities, aiming to provide older adults with a comfortable and livable environment.

Community parks are public green spaces located within urban residential neighborhoods, generally serving a radius of 500–1,000 meters and accessible on foot. They represent one of the public spaces most closely linked to the daily activities of the older adults (5–7). Compared to other urban parks, community parks have higher accessibility and are more appealing to older people with declining physical functions. Furthermore, studies have shown that contact with nature can effectively alleviate mental fatigue, and community parks provide important opportunities for such contact, thereby promoting the physical and mental well-being of older adults (8, 9). Publicly accessible community park spaces also serve as key venues for social interactions among older adults (10). Therefore, enhancing the age-friendly of community parks represents an important strategy for addressing China’s aging population and supporting the physical and mental well-being of the older adults.

Objective elements in the spatial environment of community parks—such as landscape features, facility arrangements, and environmental quality—are closely related to older adults’ actual experiences and perceptions. The needs of older adults regarding these elements are multidimensional and complex (11, 12). In terms of the physical environment, older adults prefer high-quality accessible pathways, natural vegetation, and an open park layout (13). In terms of the service facilities, park amenities such as toilets and benches, along with sports facilities primarily consisting of various types of outdoor fitness equipment, play a key role in supporting and promoting park use among older adults (14). In terms of the social-psychological dimension, older adults prefer open spaces with social interaction functions, age-appropriate social spaces, and a variety of activities, which are attractive to older adults when visiting parks (15). Therefore, assessing older adults’ subjective perceptions and satisfaction regarding the age-friendly of community park environments can provide a better understanding of their complex needs and guide the renovation and design of age-friendly community parks.

Evaluation methods are crucial for assessing the age-friendly of community parks. Traditional studies on age-friendly have largely focused on age-friendly design and renovation, while research on evaluation systems from the older adults’ perspective remains relatively limited, and systematic assessment standards have yet to be established (16–18). Some scholars have employed methods such as the Analytic Hierarchy Process (AHP), Fuzzy Comprehensive Evaluation, and Structural Equation Modeling (SEM) to assess the age-friendly of community parks. For instance, Ren (19) applied the AHP method to determine indicator weights and used a fuzzy comprehensive evaluation method to identify specific renovation priorities and scheduling for age-friendly transformations of community public spaces. However, this approach did not resolve the issue of subjectivity in weight determination (19). Lak et al. (20) employed SEM to analyze the needs of older adults in outdoor environments, but the model faced challenges in handling multi-objective decision-making problems. In recent years, the coupled Entropy Weight Method–TOPSIS model has attracted widespread attention from researchers due to its ability to integrate subjective and objective weights, perform dynamic rankings, and effectively address complex system evaluation problems (21, 22). However, few studies have applied this approach to evaluating public spaces from the perspective of older people.

Building on this, and to address gaps in previous research, this study focuses on community parks in the core area of Beijing and, from the perspective of older adult users, develops an evaluation index system encompassing five dimensions: accessibility, security, comfort, functional diversity, and social supportive. And we employed an integrated AHP–Entropy Weight–TOPSIS to conduct an empirical assessment. This study set the following objectives: (1) to develop a systematic evaluation index system for community park age-friendly by integrating expert opinions and older adult questionnaire data; (2) to identify the key influencing mechanisms of community park age-friendly from both subjective and objective perspectives; and (3) to quantify and comparatively analyze the age-friendly of community parks in the core area of Beijing, and to propose optimization strategies and planning recommendations for age-friendly park design.

## 2 Study site and method

### 2.1 Research location

This study focuses on community parks in the core area of Beijing. The core area of Beijing primarily refers to Dongcheng District and Xicheng District, which together form the capital’s core functional zone and are typical examples of high-density urban areas. It is also one of the areas with the most pronounced aging problem in Beijing. According to the Seventh National Population Census of China, the proportion of residents aged 60 and above in the core functional area of Beijing exceeds one-fourth of the total population. On this basis, this study conducted field investigations and comprehensive analyses of community parks in Beijing’s core area, and selected those with relatively favorable conditions and strong representativeness as research samples.

**Figure 1:**
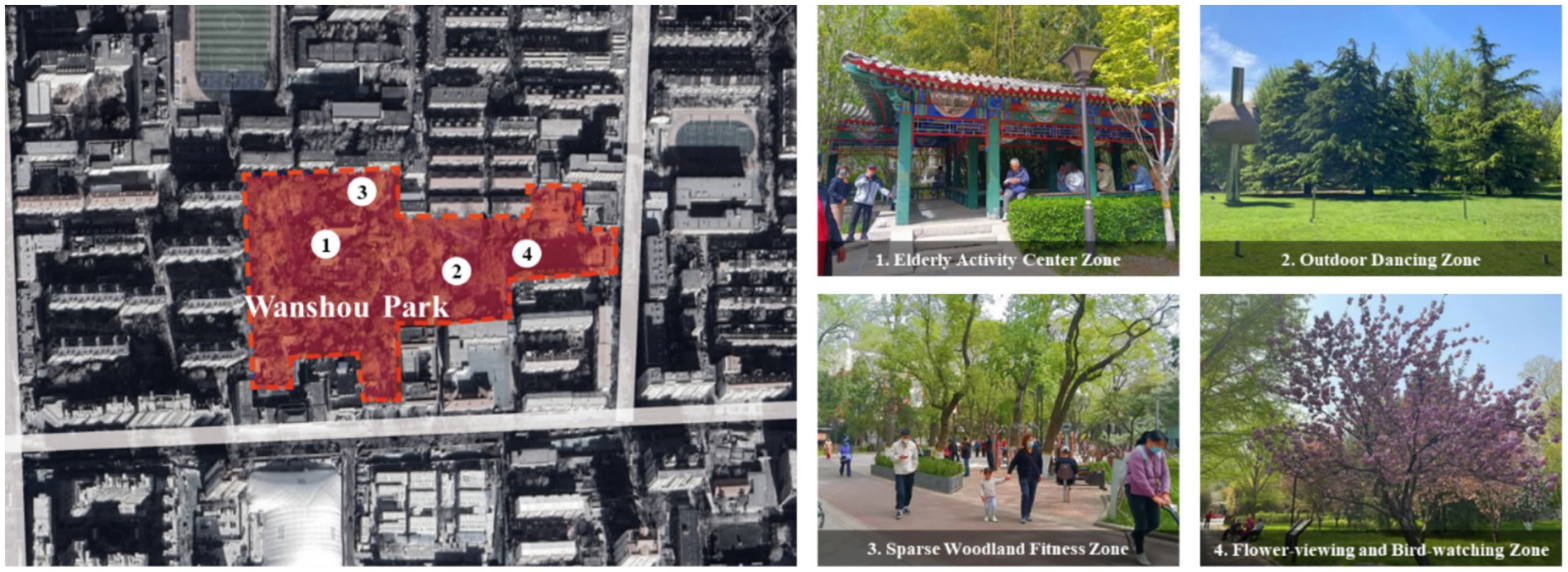
Wanshou Park.

Wanshou Park is located on the north side of Baizhifang East Street in Xicheng District, Beijing, covering an area of approximately 5.1 hectares. It is a community park themed around “filial piety” and “longevity.” Meanwhile, the area is home to Beijing’s first senior-friendly park. The park’s landscape is characterized by a traditional Chinese garden style, preserving Ming and Qing architectural features and integrating classical elements such as rocks, water bodies, pavilions, and corridors. Distinctive attractions include the central lake, green hill landscape, and cultural wall. The park also incorporates functional zones tailored to older adults’ activities, including an older adults’ activity center zone, an outdoor dancing zone, sparse woodland fitness zone, and flower-viewing and bird-watching zone, thereby addressing the diverse needs of older adults (Figure 1).

**Figure 2:**
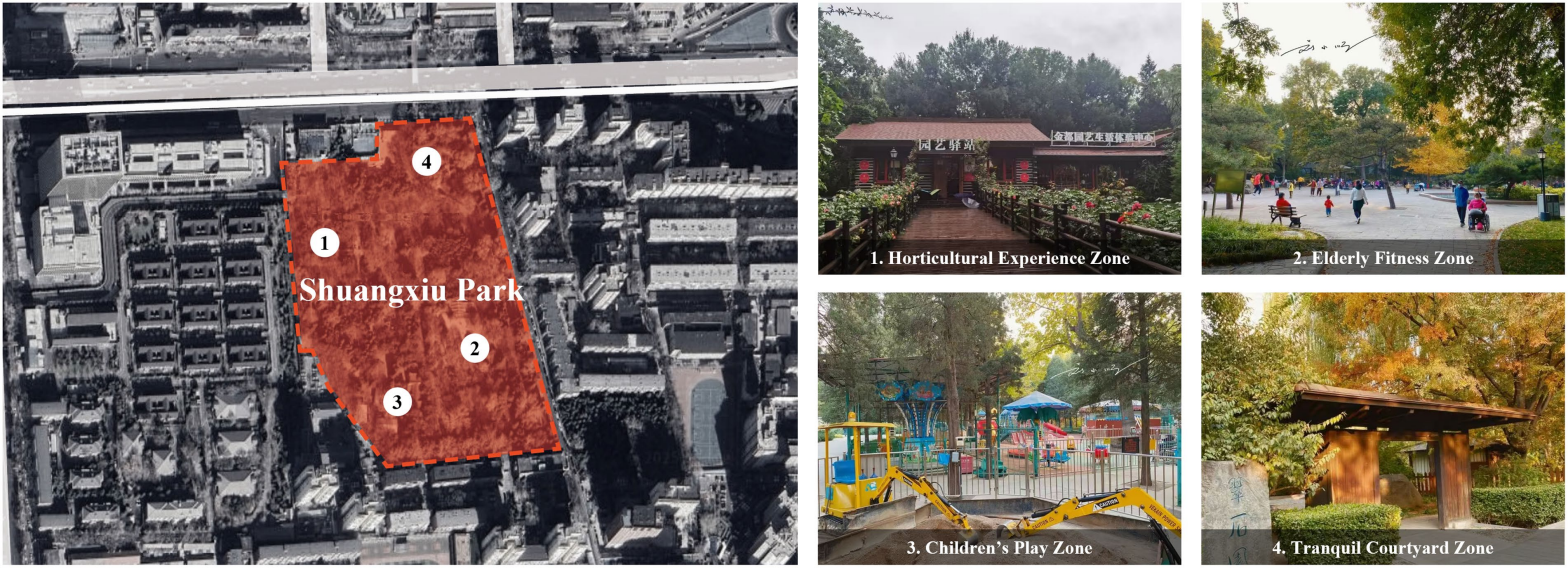
Shuangxiu Park.

Shuangxiu Park, located on North Third Ring Middle Road in Xicheng District, Beijing, covers an area of 6.4 hectares and represents a distinctive blend of Chinese and Japanese garden styles. Its landscape features traditional Chinese pavilions, a Japanese-style rock garden, meandering waterways, and diverse vegetation, forming typical Sino-Japanese characteristics with a clear spatial structure. The park is organized into functional zones including horticultural experience zone, older adults’ fitness zone, children’s play zone, and tranquil courtyard zone. Equipped with pathways, cafés, and other facilities, it provides an urban green space that integrates leisure, fitness, family activities, and nature experiences for the public (Figure 2).

**Figure 3:**
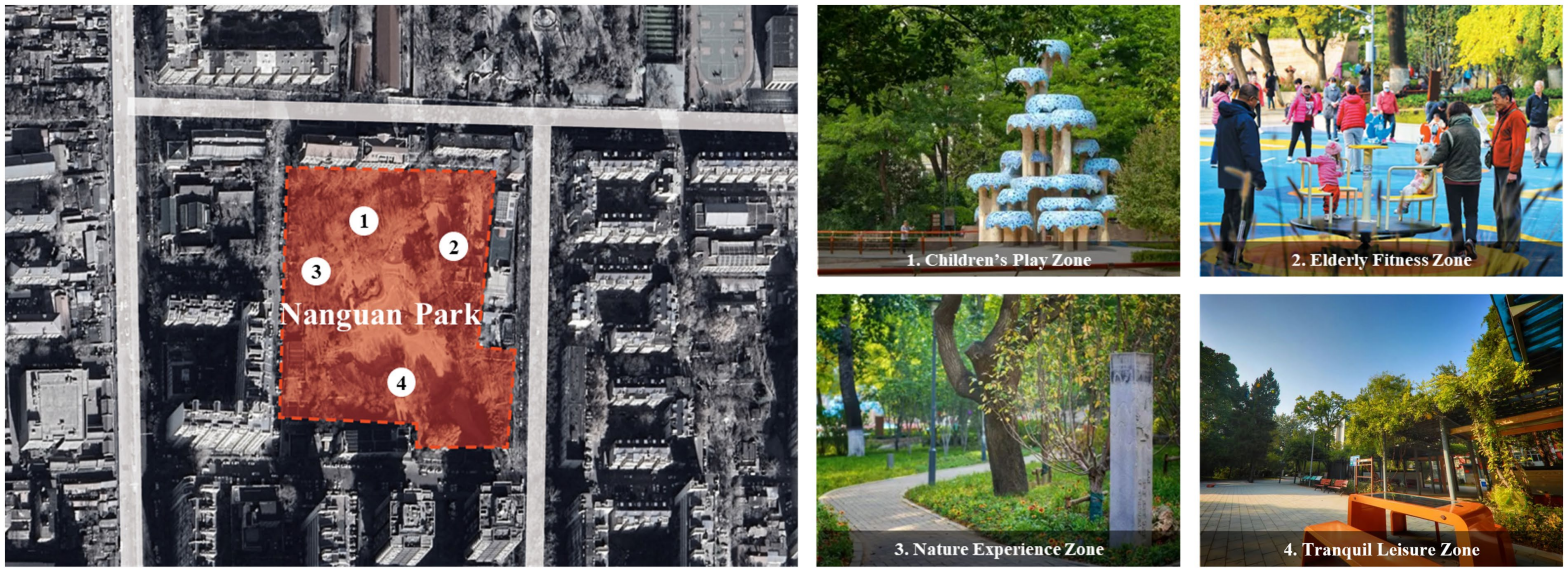
Nanguan Park.

Nanguan Park, located in Dongcheng District of Beijing and covering an area of approximately 2.9 hectares, is the city’s first urban park to integrate the concept of zero-carbon sustainability with age-friendly design. The park features a modern green landscape where technology is made visible and harmoniously blended with nature. It incorporates multiple low-carbon technologies, including solar-powered lighting and rainwater harvesting, while utilizing native and drought-tolerant plant species to create a multi-layered, evergreen ecological atmosphere. The park is organized into clearly defined functional zone, including children’s play zone, older adults’ fitness zone, nature experience zone, and tranquil leisure areas. Equipped with exhibition halls, science education pavilions, and convenient public facilities, the park achieves an organic integration of ecology, recreation, and science popularization (Figure 3).

### 2.2 Establishment of an evaluation system

This study establishes a multidimensional indicator system for evaluating the age-friendly of community parks through a process of field investigation, literature review, expert screening, and statistical validation. First, detailed field investigations were conducted at the study sites to collect extensive image data. By reviewing a large body of literature and integrating the field survey results, relevant factors influencing the assessment of community park age-friendly were preliminarily summarized.

In the expert screening stage, a total of eight experts were invited to evaluate and screen the assessment factors. As the evaluation system for age-friendly community parks is closely related to their spatial environmental elements (11), and the spatial environmental elements and their design of community parks fall within the broader domain of urban design, we therefore selected four experts with professional backgrounds in urban design. Meanwhile, following the principles of relevance to the research topic and professional coverage, we further refined the impact of community park spatial environments on older adults, and selected an additional four experts covering the fields of environmental design, architectural design, and psychology from the perspectives of environmental design, public service facilities, and environmental psychology. These scholars all have extensive research and practical experience in age-friendly design within their respective professional backgrounds. Detailed information on the backgrounds and relevant work experience of the evaluating experts can be found in Supplementary Table S1.

Regarding the evaluation method, we employed online questionnaires, which were distributed to the experts in two rounds: the first round was distributed on December 6, 2024, with all questionnaires collected by December 14, 2024; the second round was distributed on December 18, 2024, and collected by December 22, 2024. Field survey image data were provided as supplementary references to support the expert evaluations. After two rounds of Delphi consultation, the coordination coefficient (Kendall’s W) for expert opinions reached 0.824 for the primary and 0.811 for the secondary indicators, indicating strong consensus. The primary indicators were ultimately confirmed as accessibility, safety, comfort, functional diversity, and social support. The conceptual definitions of each indicator are detailed as Table 1 follows:

**Table 1:** Assessment elements and related concept explanations.

Primary indicator	Secondary indicator	Conceptual explanation
B1: accessibility	B11: spatial accessibility	Proximity and integration with urban surroundings
	B12: path accessibility	Safety and convenience of walking paths
	B13: message accessibility	Clarity and visibility of signage and wayfinding
	B14: accessibility to public facilities	Ease of access to toilets, benches, fountains
B2: security	B21: physical environment security	Safety of terrain, lighting, and surroundings
	B22: emergency safety and security	Availability of alarms and emergency response
	B23: facility security design	Safe design of handrails, pavements, structures
	B24: environmental security awareness	Security signs and public safety reminders
B3: comfort	B31: noise control and environmental tranquility	Control of noise levels and creation of quiet zones
	B32: visual environment comfort	Pleasant scenery, color, and visual harmony
	B33: microclimate regulation	Shade, ventilation, and thermal comfort
	B34: ageing of leisure facilities	Safety and usability of aging leisure equipment
	B35: psychological healing environment	Presence of soothing, restorative natural settings
B4: functional diversity	B41: cultural, educational and recreational functions	Availability of cultural, learning, and leisure activities
	B42: types of ageing-friendly fitness services	Variety of fitness options suitable for older adults
	B43: seasonal adaptation services	Facilities adapted to seasonal weather changes
	B44: intelligent service facilities	Smart screens, health monitoring, emergency systems
B5: social supportive	B51: social space design	Spaces that support communication and group activity
	B52: community activities organization	Frequency and richness of organized public events
	B53: community support networks	Presence of volunteers or peer support systems
	B54: space for intergenerational integration and interaction	Areas promoting interaction between age groups

**Figure 4:**
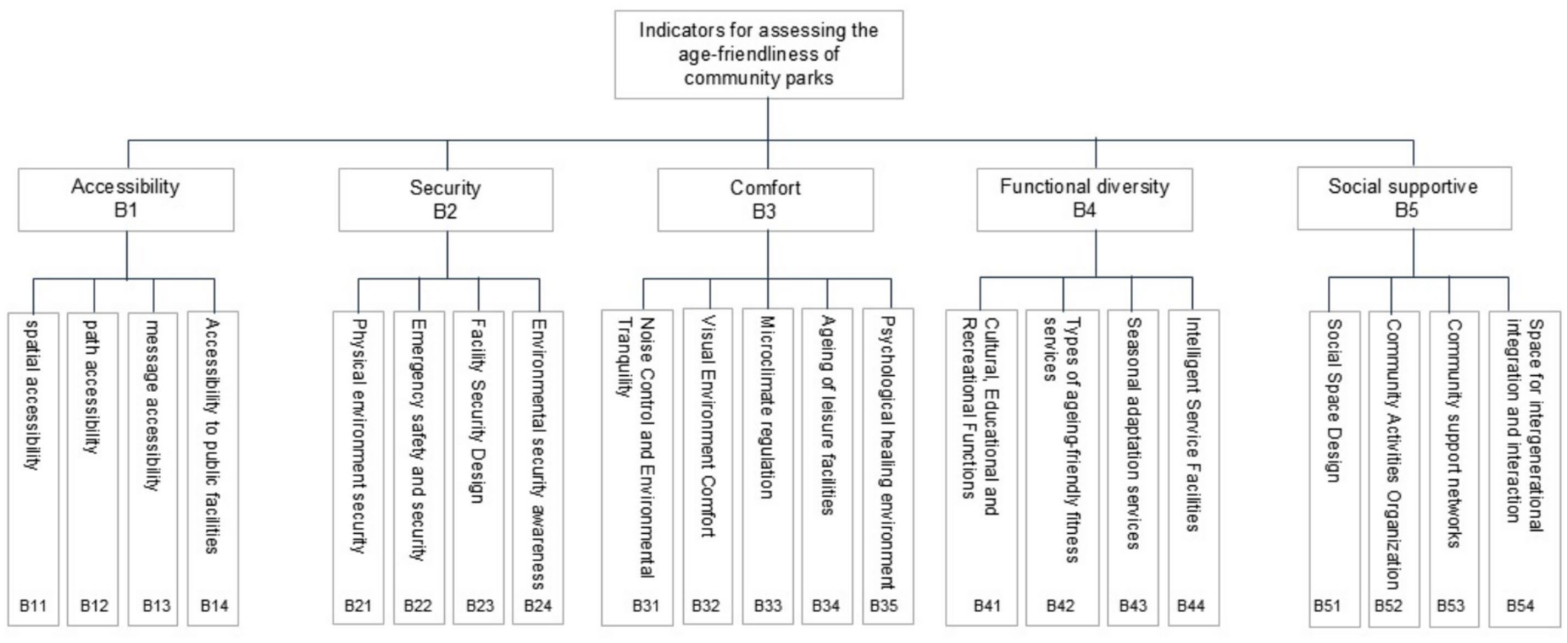
Evaluation system for aging-friendly community parks.

Using the AHP (Analytic Hierarchy Process), five first-level indicators and 21 s-level indicators were identified for the age-friendly nature of community parks, as Figure 4 follows:

(1) Accessibility: Due to the limited mobility of older adults, accessibility is a fundamental prerequisite for their use of community parks and other public spaces (23, 24). Spatial accessibility (B11) refers to a park’s geographic location and spatial distance, which are primary considerations for older adults when selecting a park (25). Path accessibility (B12) pertains to the accessibility of internal park pathways and the presence of barrier-free designs, directly affecting older adults’ activity experiences and serving as a key factor influencing park use (26). Information accessibility (B13) emphasizes the importance of legible signage, as wayfinding becomes increasingly difficult with age and declining cognitive abilities; clear, systematic, and consistently available signs significantly assist older adult visitors in navigating the park (27). Accessibility of public facilities (B14) indicates that conveniently located and well-equipped facilities exert significant positive impact on older adults’ park activities (28).
(2) Safety: Older adults generally have weaker physical functions, which increases their demand for environmental safety. Therefore, safety in community parks is a necessary precondition for ensuring that older adults can engage in activities comfortably and with confidence (29). Physical environmental safety (B21) represents the most fundamental element of site security (30). Emergency safety assurance (B22) refers to measures such as first-aid service points and safety alarm systems, which can provide psychological reassurance for older adults (31). Facility safety design (B23) emphasizes that facilities should avoid sharp edges and excessive elevation changes, while ensuring adequate lighting, thereby effectively improving older adults’ user experience (32). Environmental safety perception (B24) highlights the role of improved monitoring facilities and strengthened patrol supervision in creating a safe atmosphere and enhancing users’ sense of security (33).
(3) Comfort: Comfort is an important factor enabling older adults to use and enjoy spatial environments for extended periods (34). Noise control and environmental quietness (B31) and visual comfort (B32) are critical since excessive noise and chaotic colors may increase negative emotions, whereas auditory and visual comfort can enhance psychological tranquility, reduce stress, and create a pleasant environment (35). Microclimate regulation (B33) can influence human comfort perception within a specific microenvironment by regulating local factors such as temperature and humidity (36). Age-friendly design of recreational facilities (B34) emphasizes that age-appropriate designs can significantly improve older adults’ willingness to use them and enhance their overall experience (17). Therapeutic environments (B35) highlight the role of natural healing elements, such as water bodies and gardens, in providing psychological pleasure and comfort (37).
(4) Functional diversity: Diverse functions can provide older adults with a variety of activity venues and forms, thereby meeting their heterogeneous activity needs (12). Cultural, educational, and recreational functions (B41) enable participation in activities that offer opportunities for emotional release, alleviation of loneliness, reinforcement of self-identity, and social integration within the urban environment (38). Age-friendly fitness services (B42) encompass a variety of age-appropriate facilities that accommodate diverse exercise demands, effectively enhancing older adults’ physical health (39). Seasonal adaptation services (B43) highlight the importance of providing services and facilities that respond to seasonal and temperature variations, such as sunshades and heating equipment, to support diverse activities (40). Smart service facilities (B44) utilize intelligent technologies to enrich activity experiences for older adults while improving safety and convenience (41).
(5) Social support: Having close friends who provide emotional support is crucial for older adults’ psychological well-being and quality of life, and community parks serve as key venues for such interactions (42). Social space design (B51) provides important settings for social interaction among older adults, and the spatial layout can significantly influence their frequency of use (43). Support for community-organized activities (B52) includes group-based activities, such as square dancing, choirs, and Tai Chi, which contribute to enhancing older adults’ mental health and sense of social participation (44). Community support networks (B53) play a vital role in promoting healthy aging, exerting a stronger influence than family or friends; examples include activity promotion and mutual aid within community parks (45). Intergenerational interaction activities (B54) offer spaces for parent–child and other intergenerational activities, effectively strengthening community cohesion and improving older adults’ psychological health (46).

### 2.3 Data statistics and analysis

By combining field surveys in Beijing with a literature review, we developed a set of 21 indicators. To calculate the combined weights of these indicators from both subjective and objective perspectives, evaluation data were collected from older adult users in three community parks via questionnaires, and the Entropy Weight Method was subsequently applied to determine objective weights. Although the questionnaire data reflect the subjective perceptions of older adults, when supported by a sufficiently large sample, they can objectively represent the actual performance of each park’s spatial environment across different characteristic dimensions (47).

Based on the developed indicators, we designed a questionnaire and distributed it across the three community parks included in the study. The distribution was conducted in two stages: the first stage involved a preliminary experiment on February 10, 2025, and the second stage involved the main survey from February 12 to 15, 2024, spanning a total of 5 days. The questionnaires were administered in person. Considering that the respondents were older adults aged 60 and above, some faced difficulties in filling out the forms due to declining vision; thus, surveyors provided assistance by recording their verbal responses. Initially, 30 questionnaires were distributed at Wanshou Park for the pilot survey. Subsequently, 110 questionnaires were distributed at each of the three parks—Wanshou Park, Shuangxiu Park, and Nanguan Park—resulting in 105, 100, and 104 valid responses, respectively. In total, 330 questionnaires were distributed, with 309 valid responses collected, yielding an effective response rate of 93.64%. Among the respondents, 37.54% were male and 62.46% were female, with 89.64% aged between 60 and 80 years. Most respondents had an education level of high school or vocational college, a monthly income of 5,000–10,000 RMB, and lived with their spouse, accounting for 57.60, 58.90, and 79.29%, respectively. Detailed demographic information of the respondents is presented in Supplementary Tables S2–S4.

The Likert 5-point scale method was employed to measure overall satisfaction with community parks among older adults. Each factor in the evaluation system was assigned values of 5.0, 4.0, 3.0, 2.0, and 1.0 on a 5-point scale, corresponding to the rating categories of “very satisfied,” “fairly satisfied,” “neutral,” “not very satisfied,” and “very dissatisfied.” The older adult respondents scored based on their perception of the friendliness of community parks. The importance and relevance of the factors were positively correlated with the scores given by the older adult respondents; the higher the score, the higher the importance and relevance. The questionnaires were distributed at three community parks in the core area of Beijing and distributed to eligible individuals by members with professional knowledge. The detailed content of the questionnaire is as follows (Table 2).

**Table 2:** Quantitative evaluation index system for the age-friendliness of community parks.

Indicator code	Evaluate accessibility	Rated on a 1-to-5 scale
B11	The community park is conveniently accessible and located within a 15-min walking distance from my residence.	☐1 ☐2 ☐3 ☐4 ☐5
B12	The internal pathways of the park are smooth, safe, and accessible for both walking and wheelchair use.	☐1 ☐2 ☐3 ☐4 ☐5
B13	The signage and wayfinding systems inside and outside the park are clear, visible, and easy to identify.	☐1 ☐2 ☐3 ☐4 ☐5
B14	Supporting facilities such as public restrooms, drinking fountains, and resting areas are easily and conveniently accessible	☐1 ☐2 ☐3 ☐4 ☐5
B21	The ground surface is well maintained, free of elevation-related fall risks, and equipped with adequate protective barriers.	☐1 ☐2 ☐3 ☐4 ☐5
B22	The park is equipped with emergency facilities such as AEDs, emergency alarm systems, and emergency contact channels.	☐1 ☐2 ☐3 ☐4 ☐5
B23	Fitness equipment, seating, and handrails in the park are ergonomically designed, safe and stable, with smooth and rounded edges.	☐1 ☐2 ☐3 ☐4 ☐5
B24	The park provides a strong sense of environmental security through adequate public safety, regular security patrols, and a well-functioning surveillance system	☐1 ☐2 ☐3 ☐4 ☐5
B31	The park provides a quiet and peaceful environment	☐1 ☐2 ☐3 ☐4 ☐5
B32	The lighting system in the park provides soft and comfortable illumination in terms of brightness and color tone.	☐1 ☐2 ☐3 ☐4 ☐5
B33	The park offers effective microclimate regulation through shade trees, well-planned greenery, and misting or cooling systems.	☐1 ☐2 ☐3 ☐4 ☐5
B34	The park provides sufficient resting areas, and the seating facilities are designed to accommodate the physical needs of older adults.	☐1 ☐2 ☐3 ☐4 ☐5
B35	The design of natural elements such as landscapes, plant arrangements, and water features contributes to emotional improvement and stress relief.	☐1 ☐2 ☐3 ☐4 ☐5
B41	The park is equipped with fitness equipment, walking trails, and rehabilitation areas that accommodate the physical needs of people across different age groups.	☐1 ☐2 ☐3 ☐4 ☐5
B42	The park includes cultural and recreational facilities such as handicraft activity spaces, chess pavilions, and art exhibition corridors.	☐1 ☐2 ☐3 ☐4 ☐5
B43	The park provides seasonal adaptation measures such as shading in summer and windbreaks or heating facilities in winter.	☐1 ☐2 ☐3 ☐4 ☐5
B44	The park is equipped with intelligent facilities such as smart guide screens, health monitoring systems, emergency call devices, and interactive fitness instruction displays.	☐1 ☐2 ☐3 ☐4 ☐5
B51	The park provides satisfactory social spaces for communication and leisure activities such as playing Go	☐1 ☐2 ☐3 ☐4 ☐5
B52	The park regularly organizes group activities such as handicraft workshops or volunteer programs that promote social engagement.	☐1 ☐2 ☐3 ☐4 ☐5
B53	There are organizers, volunteers, or neighborhood networks that support social interaction and mutual assistance among older adults in the park.	☐1 ☐2 ☐3 ☐4 ☐5
B54	The park provides satisfactory intergenerational spaces, such as parent–child activity areas, that facilitate interaction between older adults, their children, and grandchildren.	☐1 ☐2 ☐3 ☐4 ☐5

### 2.4 Evaluation method

This paper adopts a combination of subjective and objective weighting methods. It combines the subjective experience of AHP with the objective data-driven approach of entropy weighting to balance qualitative and quantitative factors in decision-making. AHP is used to clarify the decision-making framework through hierarchical decomposition, while entropy weighting dynamically adjusts the weights, and TOPSIS responds to data changes. The combination of subjective and objective weighting reduces dependence on expert subjectivity and enhances the credibility of the results. The entropy weight method automatically selects high-information-content indicators, while TOPSIS rapidly completes large-scale scheme ranking and comparative analysis. This method is suitable for complex decision-making scenarios involving multiple criteria, multiple stakeholders, and heterogeneous data.

Compared with previous methods, such as fuzzy comprehensive evaluation and gray correlation, this paper introduces the entropy weight method based on expert opinions to ensure the objectivity of the weights, forming a qualitative and quantitative combined indicator weight calculation method, which effectively increases the reliability of the data in the weight calculation process.

**Figure 5:**
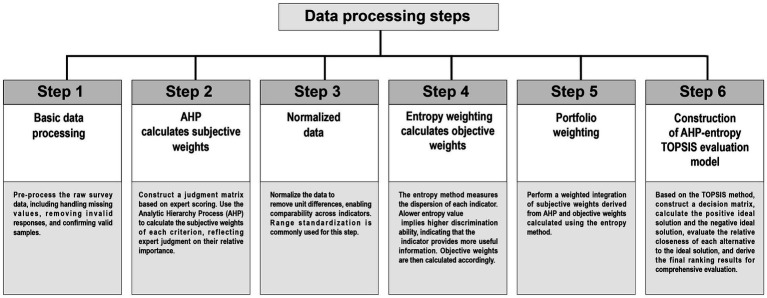
Data processing steps.

Therefore, this paper combines the existing subjective evaluation methods with the entropy weight-TOPSIS method for calculating weights to form an AHP–Entropy Weight–TOPSIS evaluation method that combines subjective and objective values. This method can balance and objectively combine expert opinions and user data to form an evaluation system for the aging-friendly design of community parks (Figure 5).

#### 2.4.1 AHP calculation of subjective indicator weights

The Analytic Hierarchy Process (AHP) is a multi-criteria decision-making method proposed by American operations researcher Thomas L. Saaty in the 1970s. Its core idea is to decompose complex problems into hierarchical structures, quantify subjective judgments using pairwise comparison matrices, determine the weights of each factor through mathematical calculations, and ultimately comprehensively evaluate the priority of alternative options (48). AHP can effectively integrate complex, multi-dimensional, and multi-stakeholder requirements through hierarchical simplification. Converting decision-makers subjective judgments into quantitative data using a 1–9 scale achieves the integration of qualitative and quantitative analysis (49).

1. Construct a hierarchical model with the following levels from top to bottom: target level, first-level indicators, and second-level indicators.
2. Construct a judgment matrix using a 9-point scale. The n-order judgment matrix is as follows (as shown in Equation 1):

$$A_{n\times n}=[\begin{array}{cccc} a_{11} & a_{12} & a_{1.} & a_{1n}\\ a_{21} & a_{22} & a_{2..} & a_{2n}\\ a_{..} & a_{..} & a_{..} & a_{..}\\ a_{n1} & a_{n2} & a_{n..} & a_{\mathrm{nn}} \end{array}]$$ (1)

3. The geometric mean method is used to combine the expert matrices to obtain a single integrated matrix. The calculation formula is as follows (as shown in Equation 2):

$$\bar{A}={\left(\prod_{k=1}^{m}a_{\mathrm{ij}}^{k}\right)}^{\frac{1}{m}}$$ (2)

4. Calculate the relative weights of the judgment matrix using the following formula (as shown in Equation 3):

$$W_{i}=\frac{{\left(\prod_{j=1}^{n}a_{\mathrm{ij}}\right)}^{\frac{1}{n}}}{\sum_{i=1}^{n}{\left(\prod_{j=1}^{n}a_{\mathrm{ij}}\right)}^{\frac{1}{n}}},i=1,2,3..,n$$ (3)

After calculating the relative weights, the consistency of the matrix was tested. A consistency ratio (CR < 0.1) indicates that the judgment matrix has good consistency. The detailed formulas for the consistency test of the judgment matrix are provided in Supplementary material.

#### 2.4.2 Calculating objective weights using the entropy weight method

Based on the evaluation data collected from older adult users through the questionnaire survey, objective weights were determined by calculating the information entropy of each indicator, which measures the degree of dispersion in the data distribution. The smaller the entropy value, the greater the information content of the indicator and the higher the weight. Based on the weights determined by the entropy weighting method, a standardized decision matrix is constructed, and the Euclidean distance between each scheme and the positive and negative ideal solutions is calculated. The schemes are then ranked according to their relative proximity (50).

(1) Establish an evaluation matrix.

When constructing multiple indicator matrices, assume that there are a evaluation objects and b evaluation indicators, and x_ij_ is the data corresponding to the jth evaluation indicator under the ith evaluation object (i = 1, 2, …, m; j = 1, 2, …, n). Form the original data matrix (as shown in Equation 4):


$$X_{\mathrm{ij}}=[\begin{array}{cccc} x11 & x12 & \cdots & x1n\\ x21 & x22 & \cdots & x2n\\ \vdots & \vdots & \cdots & \vdots \\ \mathrm{xm}1 & \mathrm{xm}2 & \cdots & \mathrm{xmn} \end{array}]$$ (4)

(2) Standardized data processing.

Since indicators of different natures can have a significant impact on the results, it is necessary to eliminate the influence of dimensions during the calculation process and normalize the data. Since all indicators in this study are positively oriented, the following processing procedure was applied:

The treatment of positive indicators is as follows (as shown in Equation 5):


$$y_{\mathrm{ij}}=\frac{x_{\mathrm{ij}}-\min(x_{j})}{\max(x_{j})-\min(x_{j})}$$ (5)

To ensure the validity of the standardized indicators, add 0.0001 to the results.

(3) Defining standardized values.

Calculate the weight of the nth indicator for i evaluation objects (as shown in Equation 6):


$$P_{\mathrm{ij}}=\frac{y_{\mathrm{ij}}}{\sum_{i=1}^{m}y_{\mathrm{ij}}}$$ (6)

(4) Calculate entropy value (as shown in Equation 7):

$$e_{j}=-\frac{1}{\mathrm{lnm}}\sum_{i=1}^{m}p_{\mathrm{ij}}\ln p_{\mathrm{ij}}\;$$ (7)

(5) Calculate the degree of variation in indicators.

Calculate the degree of variation of the jth indicator as follows (as shown in Equation 8):


$$g_{j}=1-e_{j}$$ (8)

(6) Calculate indicator weights.

Calculate the weight of the jth indicator (as shown in Equation 9):


$$w_{j}=\frac{g_{j}}{\sum_{j=1}^{n}g_{j}}$$ (9)

#### 2.4.3 Combination weight calculation

The combination method is constructed as follows:


$$W_{x}=\alpha \;\mathrm{Wi}+(1-\alpha )\mathrm{Wj}\;(\text{where}\;\alpha =0.5)$$

$W_{i}$ represents subjective weight, $W_{j}$ represents objective weight value, $W_{x}$ represents the combined weight of indicator x.

#### 2.4.4 TOPSIS evaluation model

(1) Calculate the comprehensive score (as shown in Equation 10):

$$z_{\mathrm{ij}}=w_{x*}y_{\mathrm{ij}}$$ (10)

(3) Determine positive and negative ideal solutions and calculate proximity distance (as shown in Equations 11,12):

Positive ideal solution $Z^{+}$ :


$$\begin{array}{l} Z^{+}=(Z_{1}^{+},Z_{2}^{+},\cdots ,Z_{n}^{+}),\\ \;Z_{j}^{+}=\max(W_{x}Z_{1j},W_{x}Z_{2j},\cdots ,W_{x}Z_{\mathrm{mj}})j=1,2\cdots ,n. \end{array}$$ (11)

Negative ideal solution $Z^{-}$ :


$$\begin{array}{l} Z^{-}=(Z_{1}^{-},Z_{2}^{-},\cdots ,Z_{n}^{-}),\\ Z_{j}^{-}=\min(W_{x}Z_{1j},W_{x}Z_{2j},\cdots ,W_{x}Z_{\mathrm{mj}})\;j=1,2\cdots ,n \end{array}$$ (12)

(3) Calculate the Euclidean distance between the positive and negative ideal solutions (as shown in Equations 13,14):

$$D_{i}^{+}=\sqrt{\sum_{j=1}^{n}{\left[(Z_{\mathrm{ij}}-Z_{j}^{+})\right]}^{2}}$$ (13)

$$D_{i}^{-}=\sqrt{\sum_{j=1}^{n}{\left[(Z_{\mathrm{ij}}-Z_{j}^{-})\right]}^{2}}$$ (14)

(4) Determine the proximity value $C_{i}$ between the decision scheme and the ideal solution (as shown in Equation 15):

$$C_{i}=\frac{D_{i}^{-}}{D_{i}^{-}+D_{i}^{+}}\;(i=1,2,\ldots m)$$ (15)

## 3 Results and analysis

### 3.1 AHP-entropy weighting method weighting

#### 3.1.1 Calculation results of subjective weighting indicators based on AHP

For each expert, a separate judgment matrix was constructed and subjected to a consistency test. All matrices had CR values below 0.1, indicating that they met the consistency requirement (Supplementary Tables S5–S10). Subsequently, the judgment matrices that passed the consistency test were aggregated using the geometric mean method to obtain a comprehensive judgment matrix. Based on the results of the calculation of the integration matrix and weights based on various indicators, the weights between indicators at each level are summarized as follows (Table 3).

**Table 3:** Weight summary table based on AHP analysis.

Goal level	Primary indicators	Relative weight W1	Secondary indicators	Relative weight W2	Combined weight W1*W2
Community Park Age-Friendly Evaluation A	B1: accessibility	0.2244	B11 spatial accessibility	0.1903	0.0427
			B12 path accessibility	0.3808	0.0855
			B13 message accessibility	0.2150	0.0482
			B14 accessibility to public facilities	0.2138	0.0480
	B2: security	0.2461	B21 physical environment security	0.2631	0.0647
			B22 emergency safety and security	0.2602	0.0640
			B23 facility security design	0.2159	0.0531
			B24 Environmental Security Awareness	0.2608	0.0642
	B3: comfort	0.2127	B31 noise control and environmental tranquility	0.1681	0.0358
			B32 visual environment comfort	0.1613	0.0343
			B33 microclimate regulation	0.1253	0.0267
			B34 ageing of leisure facilities	0.3599	0.0766
			B35 psychological healing environment	0.1854	0.0394
	B4: functional diversity	0.1615	B41 cultural, educational and recreational functions	0.4143	0.0669
			B42 types of ageing-friendly fitness services	0.3223	0.0521
			B43 seasonal adaptation services	0.1209	0.0195
			B44 intelligent service facilities	0.1425	0.0230
	B5: social supportive	0.1553	B51 social space design	0.4658	0.0723
			B52 community activities organization	0.1539	0.0239
			B53 community activities organization	0.1959	0.0304
			B54 space for intergenerational integration and interaction	0.1843	0.0286

#### 3.1.2 Calculation results of objective indicators based on entropy weight method

We first conducted reliability and validity analyses of the collected questionnaire data using Cronbach’s alpha, the Kaiser-Meyer-Olkin (KMO) test, and Bartlett’s test of sphericity. The results confirmed that the questionnaire data exhibited satisfactory reliability and validity, indicating that the data are reasonably robust. Detailed results are presented in Supplementary Tables S11–S12.

We standardized the data based on the average scores of each park in the questionnaire for each indicator and used the entropy method to determine the weights of each indicator. Finally, we obtained the entropy values, coefficients of variation, and weights (Table 4).

**Table 4:** Summary table of indicator entropy values, difference coefficients, and weights.

Secondary indicator	Entropy value	Coefficient of variation	Weight
B11 spatial accessibility	0.6132	0.3868	0.0328
B12 path accessibility	0.0019	0.9981	0.0846
B13 message accessibility	0.4563	0.5437	0.0461
B14 accessibility to public facilities	0.6132	0.3868	0.0328
B21 physical environment security	0.0019	0.9981	0.0846
B22 emergency safety and security	0.5126	0.4874	0.0413
B23 facility security design	0.5126	0.4874	0.0413
B24 environmental security awareness	0.5800	0.4200	0.0356
B31 noise control and environmental tranquility	0.5800	0.4200	0.0356
B32 visual environment comfort	0.5800	0.4200	0.0356
B33 microclimate regulation	0.5800	0.4200	0.0356
B34 ageing of leisure facilities	0.0019	0.9981	0.0846
B35 psychological healing environment	0.5800	0.4200	0.0356
B41 cultural, educational and recreational functions	0.0019	0.9981	0.0846
B42 types of ageing-friendly fitness services	0.5800	0.4200	0.0356
B43 seasonal adaptation services	0.6314	0.3686	0.0312
B44 intelligent service facilities	0.6314	0.3686	0.0312
B51 social space design	0.0019	0.9981	0.0846
B52 community activities organization	0.5800	0.4200	0.0356
B53 community support networks	0.5800	0.4200	0.0356
B54 space for intergenerational integration and interaction	0.5800	0.4200	0.0356

#### 3.1.3 Combined weighting results

**Figure 6:**
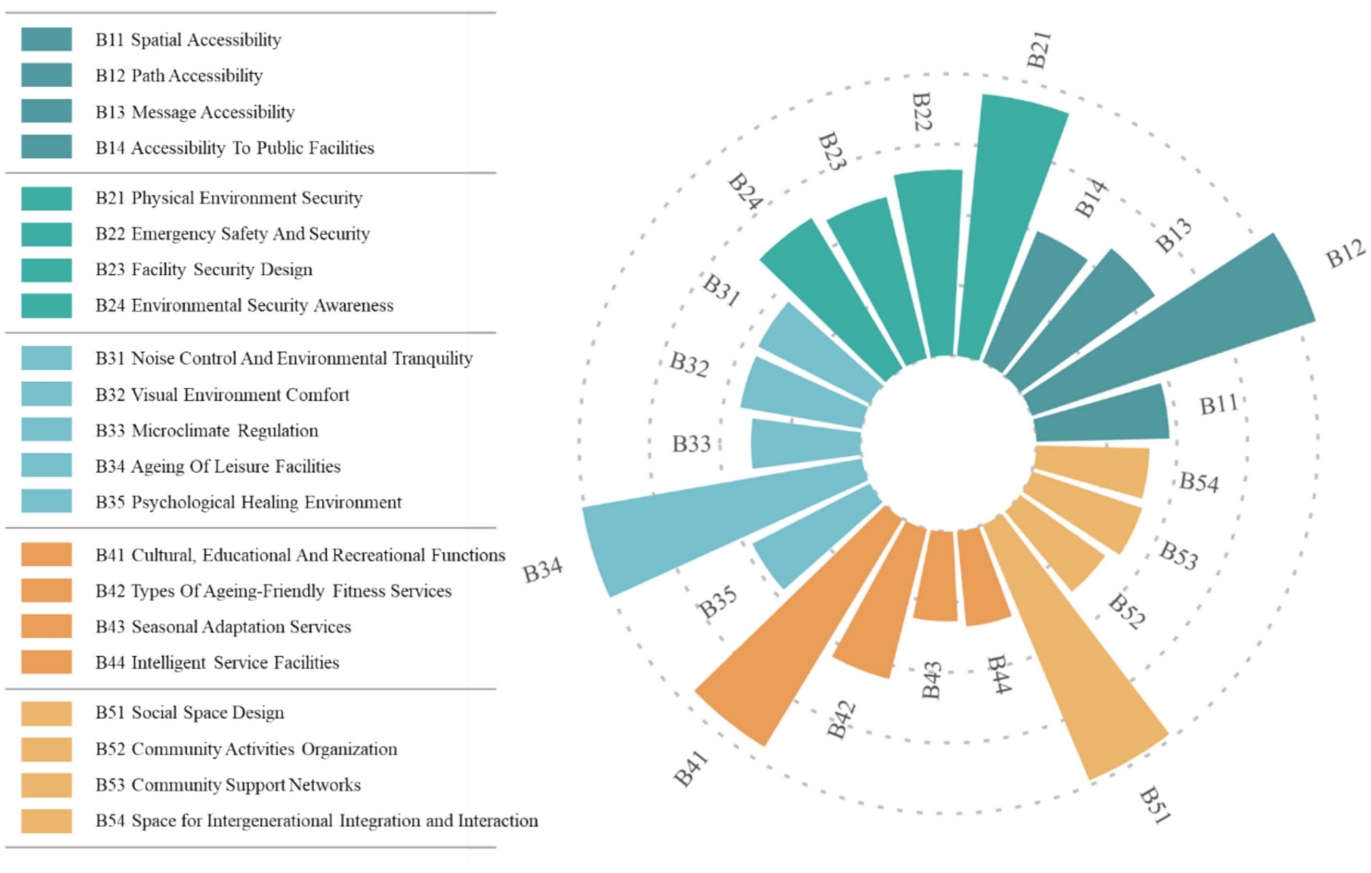
Combination weight value.

In order to avoid the shortcomings of a single weighting method and make the evaluation conclusions more scientific and reasonable, this paper combines the analytic hierarchy process and entropy weighting method to calculate the combined weights of each indicator (Figure 6). The final combined weights are as follows (Table 5).

**Table 5:** Combination weights.

Target level	Primary indicator	Secondary indicator	Combination weight
Community Park Age-Friendly Evaluation A	B1 accessibility	B11 spatial accessibility	0.0377
		B12 path accessibility	0.0850
		B13 message accessibility	0.0472
		B14 accessibility to public facilities	0.0404
	B2 security	B21 physical environment security	0.0747
		B22 emergency safety and security	0.0527
		B23 facility security design	0.0472
		B24 environmental security awareness	0.0499
	B3 comfort	B31 noise control and environmental tranquility	0.0357
		B32 visual environment comfort	0.0350
		B33 microclimate regulation	0.0311
		B34 ageing of leisure facilities	0.0806
		B35 psychological healing environment	0.0375
	B4 functional diversity	B41 cultural, educational and recreational functions	0.0757
		B42 types of ageing-friendly fitness services	0.0438
		B43 seasonal adaptation services	0.0254
		B44 intelligent service facilities	0.0271
	B5 Social supportive	B51 social space design	0.0785
		B52 community activities organization	0.0297
		B53 community support networks	0.0330
		B54 space for intergenerational integration and interaction	0.0321

### 3.2 TOPSIS evaluation model

TOPSIS (Technique for Order of Preference by Similarity to Ideal Solution) ranks and evaluates various schemes by standardizing, weighting, and calculating the distance between the evaluation object’s indicator data and the ideal solution, then determining the relative proximity. The core principle of this method is that the optimal solution should be closest to the positive ideal solution and farthest from the negative ideal solution. In this study, questionnaires were distributed to residents who met the criteria, and satisfaction levels for each indicator were rated on a scale of 1–5, yielding scores for each park across all indicators, which formed the decision matrix. Combination weights were derived by combining the AHP method (subjective) with the entropy weight method (objective), resulting in a weighted decision matrix. Finally, the distance between each park and the ideal aging-friendly state is calculated, followed by the relative proximity, resulting in an aging-friendly comprehensive score. Parks with higher relative proximity have better aging-friendly levels.

1. Based on the calculations, the comprehensive scores for each indicator in the research case are as follows (Table 6).
2. The Euclidean distance and relative proximity between the overall positive and negative ideal solutions for the three community parks are as follows. Among them, Wanshou Park has the highest relative proximity, so it has the highest satisfaction rating for aging-friendly design and is closest to the ideal state (Table 7).

**Table 6:** Comprehensive scores for each indicator.

Secondary Indicators	Wanshou Park	Shuangxiu Park	Nanguan Park
B11 spatial accessibility	0.0377	0.0000	0.0252
B12 path accessibility	0.0850	0.0000	0.0000
B13 message accessibility	0.0472	0.0000	0.0118
B14 accessibility to public facilities	0.0269	0.0000	0.0404
B21 physical environment security	0.0747	0.0000	0.0000
B22 emergency safety and security	0.0527	0.0176	0.0000
B23 facility security design	0.0472	0.0000	0.0157
B24 environmental security awareness	0.0499	0.0249	0.0000
B31 noise control and environmental tranquility	0.0357	0.0000	0.0178
B32 visual environment comfort	0.0350	0.0175	0.0000
B33 microclimate regulation	0.0311	0.0156	0.0000
B34 ageing of leisure facilities	0.0806	0.0000	0.0000
B35 psychological healing environment	0.0375	0.0000	0.0188
B41 cultural, educational and recreational functions	0.0758	0.0000	0.0000
B42 types of ageing-friendly fitness services	0.0438	0.0000	0.0219
B43 seasonal adaptation services	0.0254	0.0000	0.0254
B44 intelligent service facilities	0.0000	0.0271	0.0271
B51 social space design	0.0785	0.0000	0.0000
B52 community activities organization	0.0297	0.0149	0.0000
B53 community support networks	0.0330	0.0165	0.0000
B54 space for intergenerational integration and interaction	0.0321	0.0161	0.0000

**Table 7:** Evaluation of calculation results.

Name	Forward ideal solution distance	Backward ideal solution distance	Relative proximity	Ranking
Wanshou Park	0.0303	0.2313	0.8842	1
Shuangxiu Park	0.2170	0.0545	0.2006	3
Nanguan Park	0.2126	0.0720	0.2529	2

## 4 Discussion

### 4.1 Analysis of the mechanisms influencing age-friendly in community parks

The composite weights determine the relative importance of each indicator in influencing the age-friendly of community parks. The composite weights obtained in this study are more comprehensive and accurate compared to those derived from single subjective or objective perspectives. The results indicate that path accessibility (B12) is the most critical factor affecting satisfaction with the age-friendly of community parks, followed by ageing of leisure facilities (B34), social space design (B51), cultural, educational and recreational functions (B41), and physical environment security (B21).

First, path accessibility holds the highest weight, indicating that for older adults with reduced mobility, the continuity and barrier-free design of internal park pathways, along with clear wayfinding signage, are prerequisites for entering and using the park. This finding aligns with existing studies emphasizing that “walkability is the primary factor influencing older adults’ choice of parks,” further confirming the fundamental role of accessibility in aging societies (23, 51). Second, the importance of ageing of leisure facilities underscores the core needs of older adults in community parks—namely, rest and social interaction—which is consistent with previous research (15, 52). Numerous empirical studies have shown that well-distributed seating, sunshades, and chairs with backrests not only meet older adults’ physical recovery needs but also facilitate informal social interactions, thereby enhancing their well-being and sense of belonging (53). Third, the high weight of social space design indicates that emotional support and social interaction are indispensable components of older adults’ daily lives and represent core functions that community parks should provide. Rapid urbanization in China, characterized by large-scale population migration and the spatial separation of work and residence, has resulted in many ‘empty-nest’ older adults, who face substantial risks to their physical and mental health (54–56). In this context, thoughtfully designed social spaces can significantly enhance social interactions for these individuals, reducing feelings of loneliness and social isolation (43). For example, the provision of seating, pavilions, sunshades, and chess rooms offers platforms for social engagement and high-quality social interaction environments, markedly improving older adults’ well-being. Fourth, the cultural, educational and recreational functions occupies a significant position in the indicator system, reflecting older adults’ strong sense of competence and autonomy. Cultural learning activities and physical exercise serve as means for older adults to psychologically counteract physical aging, enabling them to experience a sense of control over their bodies (57, 58). Such activities often require a certain duration and possess engaging qualities, helping to fill older adults’ extended leisure time and making these venues particularly important. Finally, physical environmental safety (B21) is critical, as older adults with limited mobility are prone to falls due to uneven surfaces or insufficient anti-slip measures, posing serious health risks. A safe physical environment provides older adults with psychological comfort and pleasure (59).

Additionally, we found that the weights of intelligent service facilities (B44) and seasonal adaptation services (B43) were relatively low. The lower weight of smart service facilities is primarily due to older adults’ limited acceptance of new technologies and their preference for familiar routines; moreover, the learning cost associated with operating such facilities is often high, resulting in reduced attention from older adults (60). The relatively low weight of seasonal adaptation services can be attributed to the difficulty of implementing substantial seasonal adaptations in outdoor community park spaces, as well as older adults’ tendency to adjust their activities according to seasonal changes (61). Interestingly, contrary to the findings of Gough et al. (10) and Winstead et al. (62), we also observed a relatively low weight for community activities organization (B52). Unlike Western contexts, where community mutual aid organizations are common, activities among older adults in Chinese communities are largely spontaneous, with minimal formal organization or intervention. Additionally, community-organized activities often entail financial costs and diverse preferences, making them less acceptable to older adults in China (63–65).

In summary, this study reveals that older adults’ core needs in the use of community parks are concentrated in four key aspects: accessibility, recreation, social interaction, and safety, providing a clear hierarchy of priorities for future age-friendly design.

### 4.2 Assessment differences in age-friendly of community parks and optimization strategies

Based on the composite age-friendly scores of the three case study parks, Wanshou Park performed the best, whereas Shuangxiu Park and Nanguan Park showed relatively lower performance. Wanshou Park’s superior performance can be attributed to the clarity of its pathway system, the completeness of its recreational facilities, and the diversity of its social spaces, which provided a competitive advantage on the highest-weighted indicators. Nanguan Park excelled in smart facilities and cultural-educational functions—for example, the provision of digital guide screens and cultural exhibition areas enhanced educational and interactive experiences—but fell short on core indicators such as path accessibility and the quantity of recreational facilities, preventing it from achieving the top overall rating. Although Shuangxiu Park had a relatively well-maintained green environment, it lacked systematic age-friendly design; insufficient coverage of barrier-free pathways, uneven distribution of recreational facilities, and limited types of fitness equipment constrained older adults’ experiences, resulting in the lowest overall age-friendly score.

Based on these findings, the design of age-friendly community parks should prioritize several key aspects. First, site selection for the newly established community parks should emphasize connectivity with surrounding residential areas, as this directly affects older adults’ usage rates and satisfaction. For existing parks such as Shuangxiu Park and Nanguan Park, renovations should first focus on improving the pathway system. Although enhancing spatial accessibility may be constrained by the park’s location, internal circulation should be clarified. Continuous, gently sloped barrier-free paths should be added, along with tactile pavements, handrails, and wayfinding signage to ensure safe and convenient access to all functional areas. Clear navigation networks between entrances and core facilities should also be established to reduce the risk of disorientation or fatigue among older adults. Second, recreational facilities should be optimized in both quantity and layout. Seats with backrests, benches with armrests, and shaded shelters should be increased, and “rest nodes” should be provided along long pathways to create a rhythmic ‘move–rest–move’ spatial experience. Third, for social functions, multi-level social spaces should be created. Large plazas can accommodate group activities, while semi-enclosed pavilions or platforms facilitate smaller-scale interactions. Diverse social spaces can accommodate a wider range of social activities, thereby enhancing the vitality of the space. Fourth, fitness services should be tailored to the physical differences of older adults. Common light exercise equipment should be retained, while additional facilities for balance training, rehabilitation, and flexibility exercises should be provided to meet the diverse needs of older adults with varying health conditions.

Therefore, optimizing the age-friendly of community parks not only involves improving internal park facilities but should also be guided by the actual needs of older adults. By implementing systematic and differentiated design strategies, the overall quality of the space can be enhanced, thereby advancing the sustainable development of age-friendly community environments.

### 4.3 Study limitations and future perspectives

This study has several limitations and uncertainties. Although evaluating the age-friendliness of community parks in the core area of Beijing provides a certain degree of representativeness, the number of parks included is limited, and the generalizability of the findings requires further validation in other cities and regions. Future research could expand the number of questionnaires to obtain more stable and robust survey data. Furthermore, while the construction of the indicator system through field surveys, literature review, expert screening, and statistical validation is widely recognized, potential limitations remain, including subjectivity in indicator selection and the possible omission of relevant indicator dimensions.

This study applied the AHP–Entropy Weight–TOPSIS evaluation method, integrating both subjective and objective dimensions to construct an evaluation indicator system for assessing the age-friendliness of community parks in the core area of Beijing. The findings provide theoretical guidance and methodological reference for the design, assessment, management, and optimization of age-friendly community parks. However, despite the use of the Entropy Weight Method and a large sample of questionnaire data to objectively reflect the conditions of community park environments, limitations related to objectivity may still exist. Therefore, future research could develop quantitative evaluation indicators based on the objective environmental characteristics of community parks, enabling direct assessment through field surveys. Additionally, combining objective field assessment data with users’ subjective evaluations could further elucidate the mechanisms influencing the age-friendliness of community parks.

## 5 Conclusion

Quantitative assessment of the age-friendly of community parks is crucial for promoting healthy aging strategies and optimizing urban public space planning. This study examined typical community parks in the core area of Beijing and established an age-friendly evaluation indicator system through field surveys, literature review, expert screening, and statistical validation. Composite weights were determined using a combination of the AHP, the Entropy Weight Method, and the TOPSIS model was applied for comprehensive evaluation. The results indicate that path accessibility, ageing of leisure facilities, social space design, cultural, educational and recreational functions, and physical environment security are the core factors influencing age-friendly, with older adults placing particular emphasis on physical elements that directly enhance daily experiences. Significant differences in age-friendly levels were observed among the three case study parks: Wanshou Park performed the best; Nanguan Park demonstrated strengths in smart facilities and cultural-educational functions but was limited by insufficient core facilities; and Shuangxiu Park, despite its ecological foundation, lacked systematic age-friendly design. Based on these findings, this study proposes optimization strategies, including improving pathway systems, enhancing recreational and fitness facilities, creating multi-level social spaces, and integrating smart facilities with traditional park features. Compared with existing studies, this research specifically targets community parks and age-friendly, addressing a notable gap in the literature. Methodologically, the integration of AHP and the Entropy Weight Method enabled a combination of subjective and objective weighting, while the TOPSIS model ensured scientific rigor and robustness. This study provides both theoretical guidance and practical reference for the design and evaluation of age-friendly community parks, supporting evidence-based strategies to enhance older adults’ urban experiences.

## Data Availability

The original contributions presented in the study are included in the article/Supplementary material, further inquiries can be directed to the corresponding author.
