# Supplementary material for: Assessing urban community parks from an age-friendly perspective: a multi-criteria decision-making approach
Source: Front Public Health. 2025 Nov 7;13:1663359. doi: 10.3389/fpubh.2025.1663359 (PMC12634358; doi:10.3389/fpubh.2025.1663359)
Supplement: Supplementary file 1 [file Supplementary_file_1.docx]

**Supplementary material**

**Table S1.** Detailed Background Information on the Assessment Expert.

| **Number** | **Age** | **Educational background** | **Professional title** | **Research Areas** | **Institution** | **Relevant work experience** |
| --- | --- | --- | --- | --- | --- | --- |
| 1 | 37 | Doctor | Intermediate professional title | Psychology | Hospital | Extensive experience in geriatric psychology research and clinical practice. |
| 2 | 51 | Doctor | Associate Professor | Environmental Design | University | Extensive experience in age-friendly environmental design and related research. |
| 3 | 43 | Master | Senior Engineer | Urban Design | Large design institute | Led several elderly care space design projects, including implementation and user experience assessment. |
| 4 | 39 | Master | Senior Engineer | Urban Design | Large design institute | Led several elderly care space design projects, including implementation and user experience assessment. |
| 5 | 48 | Master | Senior Engineer | Architectural Design | Large design institute | Led several elderly care space design projects, including implementation and user experience assessment. |
| 6 | 32 | Master | Middle enginner | Urban Design | Large design institute | Extensive experience in age-friendly design of urban public and residential spaces. |
| 7 | 32 | Master | Middle enginner | Urban Design | Large design institute | Extensive experience in age-friendly design of urban public and residential spaces. |
| 8 | 33 | Master | Middle enginner | Architectural Design | Large design institute | Extensive experience in both the design and research of elderly residential spaces. |

**Table S2.** Basic Information of Survey Respondents in Wanshou Park.

| **Numbe** | **Gender** | **Age** | **Education level** | **Income (RMB/Month)** | **Living status** |
| --- | --- | --- | --- | --- | --- |
| 1 | Female | 60-69 | High school or vocational college | 5000-10000 | Living with spouse |
| 2 | Female | 60-69 | High school or vocational college | 5000-10000 | Living with spouse |
| 3 | Female | 60-69 | High school or vocational college | 5000-10000 | Living with spouse |
| 4 | Male | 60-69 | Bachelor’s degree | 5000-10000 | Living with spouse |
| 5 | Female | 80 and above | Junior high school and below | 10000 and above | Living alone |
| 6 | Female | 80 and above | Junior high school and below | 5000-10000 | Living with children (or spouse and children) |
| 7 | Male | 80 and above | Junior high school and below | 5000-10000 | Living with spouse |
| 8 | Male | 70-79 | Junior high school and below | 5000-10000 | Living with spouse |
| 9 | Male | 80 and above | High school or vocational college | 5000-10000 | Living with spouse |
| 10 | Male | 70-79 | High school or vocational college | 10000 and above | Living alone |
| 11 | Male | 70-79 | High school or vocational college | 5000-10000 | Living with spouse |
| 12 | Male | 70-79 | High school or vocational college | 5000-10000 | Living with spouse |
| 13 | Female | 70-79 | Bachelor’s degree | 10000 and above | Living with children (or spouse and children) |
| 14 | Male | 70-79 | High school or vocational college | 10000 and above | Living with spouse |
| 15 | Male | 70-79 | High school or vocational college | 5000-10000 | Living with spouse |
| 16 | Female | 70-79 | High school or vocational college | 10000 and above | Living with children (or spouse and children) |
| 17 | Female | 60-69 | Bachelor’s degree | 5000-10000 | Living with spouse |
| 18 | Female | 60-69 | Bachelor’s degree | 5000 and below | Living with spouse |
| 19 | Female | 80 and above | Junior high school and below | 5000-10000 | Living with children (or spouse and children) |
| 20 | Female | 80 and above | Junior high school and below | 5000-10000 | Living with children (or spouse and children) |
| 21 | Female | 80 and above | Junior high school and below | 5000-10000 | Living with spouse |
| 22 | Male | 80 and above | Bachelor’s degree | 10000 and above | Living alone |
| 23 | Male | 60-69 | High school or vocational college | 10000 and above | Living with spouse |
| 24 | Female | 70-79 | High school or vocational college | 5000-10000 | Living alone |
| 25 | Male | 70-79 | Bachelor’s degree | 5000-10000 | Living alone |
| 26 | Male | 70-79 | Bachelor’s degree | 10000 and above | Living with spouse |
| 27 | Male | 70-79 | Bachelor’s degree | 10000 and above | Living with spouse |
| 28 | Male | 80 and above | Junior high school and below | 5000-10000 | Living with spouse |
| 29 | Male | 70-79 | Junior high school and below | 5000 and below | Living with spouse |
| 30 | Female | 70-79 | Junior high school and below | 5000 and below | Living with spouse |
| 31 | Female | 70-79 | Junior high school and below | 5000-10000 | Living with spouse |
| 32 | Male | 70-79 | Junior high school and below | 5000-10000 | Living with spouse |
| 33 | Female | 70-79 | Junior high school and below | 5000-10000 | Living with spouse |
| 34 | Female | 70-79 | Junior high school and below | 10000 and above | Living alone |
| 35 | Female | 70-79 | Junior high school and below | 10000 and above | Living with children (or spouse and children) |
| 36 | Female | 60-69 | Junior high school and below | 5000-10000 | Living with spouse |
| 37 | Female | 60-69 | Junior high school and below | 5000 and below | Living with spouse |
| 38 | Female | 60-69 | High school or vocational college | 5000-10000 | Living with spouse |
| 39 | Female | 60-69 | High school or vocational college | 5000-10000 | Living with spouse |
| 40 | Male | 80 and above | Junior high school and below | 5000-10000 | Living alone |
| 41 | Female | 80 and above | High school or vocational college | 5000-10000 | Living with spouse |
| 42 | Female | 70-79 | Bachelor’s degree | 5000-10000 | Living alone |
| 43 | Female | 70-79 | Bachelor’s degree | 10000 and above | Living with spouse |
| 44 | Female | 70-79 | High school or vocational college | 10000 and above | Living with children (or spouse and children) |
| 45 | Male | 70-79 | High school or vocational college | 5000-10000 | Living with children (or spouse and children) |
| 46 | Female | 70-79 | High school or vocational college | 5000-10000 | Living with spouse |
| 47 | Female | 60-69 | High school or vocational college | 5000-10000 | Living with spouse |
| 48 | Female | 60-69 | High school or vocational college | 10000 and above | Living with spouse |
| 49 | Female | 80 and above | Bachelor’s degree | 5000-10000 | Living with children (or spouse and children) |
| 50 | Female | 80 and above | Junior high school and below | 5000 and below | Living with children (or spouse and children) |
| 51 | Male | 80 and above | Junior high school and below | 5000 and below | Living with children (or spouse and children) |
| 52 | Female | 70-79 | High school or vocational college | 5000-10000 | Living with spouse |
| 53 | Male | 80 and above | High school or vocational college | 10000 and above | Living with children (or spouse and children) |
| 54 | Female | 70-79 | High school or vocational college | 5000 and below | Living with spouse |
| 55 | Female | 70-79 | High school or vocational college | 10000 and above | Living with spouse |
| 56 | Female | 70-79 | High school or vocational college | 5000-10000 | Living with spouse |
| 57 | Male | 60-69 | Bachelor’s degree | 10000 and above | Living with spouse |
| 58 | Female | 80 and above | Junior high school and below | 5000-10000 | Living with spouse |
| 59 | Female | 60-69 | High school or vocational college | 10000 and above | Living alone |
| 60 | Male | 60-69 | High school or vocational college | 5000-10000 | Living with spouse |
| 61 | Male | 60-69 | High school or vocational college | 5000 and below | Living with spouse |
| 62 | Female | 60-69 | High school or vocational college | 5000-10000 | Living with spouse |
| 63 | Female | 60-69 | Bachelor’s degree | 5000 and below | Living with spouse |
| 64 | Male | 60-69 | High school or vocational college | 5000 and below | Living with spouse |
| 65 | Female | 60-69 | High school or vocational college | 5000 and below | Living with spouse |
| 66 | Female | 60-69 | Master’s degree or above | 10000 and above | Living with spouse |
| 67 | Female | 60-69 | High school or vocational college | 10000 and above | Living with spouse |
| 68 | Female | 60-69 | High school or vocational college | 5000-10000 | Living with spouse |
| 69 | Male | 70-79 | Bachelor’s degree | 5000-10000 | Living with spouse |
| 70 | Female | 70-79 | High school or vocational college | 5000-10000 | Living with spouse |
| 71 | Female | 70-79 | High school or vocational college | 5000-10000 | Living with spouse |
| 72 | Female | 70-79 | Junior high school and below | 10000 and above | Living with spouse |
| 73 | Female | 70-79 | High school or vocational college | 5000-10000 | Living with spouse |
| 74 | Male | 70-79 | Bachelor’s degree | 5000-10000 | Living with spouse |
| 75 | Female | 70-79 | Bachelor’s degree | 10000 and above | Living alone |
| 76 | Male | 70-79 | High school or vocational college | 5000-10000 | Living with spouse |
| 77 | Male | 70-79 | High school or vocational college | 5000-10000 | Living with spouse |
| 78 | Male | 80 and above | Master’s degree or above | 10000 and above | Living alone |
| 79 | Female | 70-79 | High school or vocational college | 10000 and above | Living with spouse |
| 80 | Female | 70-79 | High school or vocational college | 5000 and below | Living with spouse |
| 81 | Female | 80 and above | Junior high school and below | 5000 and below | Living alone |
| 82 | Male | 80 and above | Junior high school and below | 5000 and below | Living alone |
| 83 | Female | 80 and above | High school or vocational college | 5000-10000 | Living alone |
| 84 | Male | 80 and above | Junior high school and below | 5000 and below | Living with spouse |
| 85 | Male | 70-79 | High school or vocational college | 5000-10000 | Living with spouse |
| 86 | Female | 70-79 | High school or vocational college | 5000 and below | Living with spouse |
| 87 | Female | 70-79 | High school or vocational college | 5000-10000 | Living with spouse |
| 88 | Female | 70-79 | High school or vocational college | 5000 and below | Living with spouse |
| 89 | Female | 70-79 | High school or vocational college | 5000 and below | Living with spouse |
| 90 | Female | 70-79 | Bachelor’s degree | 5000 and below | Living with spouse |
| 91 | Male | 70-79 | Bachelor’s degree | 5000-10000 | Living alone |
| 92 | Female | 60-69 | Bachelor’s degree | 5000-10000 | Living alone |
| 93 | Male | 70-79 | Bachelor’s degree | 5000-10000 | Living alone |
| 94 | Male | 60-69 | Master’s degree or above | 10000 and above | Living with children (or spouse and children) |
| 95 | Female | 60-69 | Junior high school and below | 5000-10000 | Living with spouse |
| 96 | Female | 60-69 | Junior high school and below | 5000-10000 | Living with spouse |
| 97 | Female | 60-69 | Master’s degree or above | 10000 and above | Living with children (or spouse and children) |
| 98 | Female | 60-69 | Junior high school and below | 5000-10000 | Living with spouse |
| 99 | Female | 60-69 | High school or vocational college | 5000-10000 | Living with spouse |
| 100 | Male | 70-79 | Junior high school and below | 5000-10000 | Living with spouse |
| 101 | Male | 70-79 | Junior high school and below | 10000 and above | Living with spouse |
| 102 | Female | 60-69 | High school or vocational college | 10000 and above | Living alone |
| 103 | Female | 60-69 | High school or vocational college | 5000-10000 | Living with spouse |
| 104 | Female | 60-69 | High school or vocational college | 5000-10000 | Living with children (or spouse and children) |
| 105 | Female | 60-69 | High school or vocational college | 5000-10000 | Living with spouse |

**Table S3.** Basic Information of Survey Respondents in Shuangxiu Park.

| **Numbe** | **Gender** | **Age** | **Education level** | **Income (RMB/Month)** | **Living status** |
| --- | --- | --- | --- | --- | --- |
| 1 | Female | 60-69 | High school or vocational college | 5000-10000 | Living with spouse |
| 2 | Male | 60-69 | High school or vocational college | 5000-10000 | Living with spouse |
| 3 | Female | 70-79 | High school or vocational college | 5000-10000 | Living with spouse |
| 4 | Female | 70-79 | High school or vocational college | 10000 and above | Living with spouse |
| 5 | Female | 70-79 | High school or vocational college | 10000 and above | Living alone |
| 6 | Male | 60-69 | High school or vocational college | 5000-10000 | Living with spouse |
| 7 | Male | 70-79 | Junior high school and below | 5000 and below | Living with spouse |
| 8 | Female | 60-69 | High school or vocational college | 5000-10000 | Living with spouse |
| 9 | Male | 60-69 | High school or vocational college | 5000-10000 | Living with spouse |
| 10 | Female | 60-69 | Junior high school and below | 5000-10000 | Living with spouse |
| 11 | Female | 80 and above | High school or vocational college | 5000-10000 | Living with spouse |
| 12 | Male | 70-79 | High school or vocational college | 5000-10000 | Living alone |
| 13 | Female | 70-79 | Bachelor’s degree | 10000 and above | Living alone |
| 14 | Female | 70-79 | High school or vocational college | 5000-10000 | Living with children (or spouse and children) |
| 15 | Female | 60-69 | High school or vocational college | 10000 and above | Living with spouse |
| 16 | Male | 70-79 | Bachelor’s degree | 10000 and above | Living with spouse |
| 17 | Female | 70-79 | High school or vocational college | 5000-10000 | Living with spouse |
| 18 | Female | 60-69 | Bachelor’s degree | 10000 and above | Living with spouse |
| 19 | Male | 60-69 | Bachelor’s degree | 10000 and above | Living with spouse |
| 20 | Female | 60-69 | Bachelor’s degree | 10000 and above | Living with spouse |
| 21 | Male | 70-79 | Bachelor’s degree | 10000 and above | Living with spouse |
| 22 | Male | 70-79 | High school or vocational college | 10000 and above | Living with spouse |
| 23 | Female | 60-69 | High school or vocational college | 5000-10000 | Living with spouse |
| 24 | Female | 80 and above | Junior high school and below | 5000-10000 | Living with spouse |
| 25 | Male | 60-69 | High school or vocational college | 5000-10000 | Living with spouse |
| 26 | Male | 70-79 | High school or vocational college | 5000-10000 | Living with spouse |
| 27 | Female | 70-79 | High school or vocational college | 5000-10000 | Living with children (or spouse and children) |
| 28 | Male | 60-69 | Bachelor’s degree | 10000 and above | Living with spouse |
| 29 | Female | 60-69 | High school or vocational college | 10000 and above | Living with children (or spouse and children) |
| 30 | Female | 70-79 | High school or vocational college | 10000 and above | Living with spouse |
| 31 | Female | 70-79 | Junior high school and below | 5000-10000 | Living with spouse |
| 32 | Male | 70-79 | Bachelor’s degree | 5000-10000 | Living with spouse |
| 33 | Female | 80 and above | High school or vocational college | 5000-10000 | Living with spouse |
| 34 | Male | 60-69 | Master’s degree or above | 10000 and above | Living with spouse |
| 35 | Male | 60-69 | High school or vocational college | 5000-10000 | Living with spouse |
| 36 | Female | 70-79 | Bachelor’s degree | 10000 and above | Living with spouse |
| 37 | Female | 70-79 | Junior high school and below | 10000 and above | Living with spouse |
| 38 | Male | 60-69 | High school or vocational college | 5000-10000 | Living with spouse |
| 39 | Male | 60-69 | High school or vocational college | 5000-10000 | Living with spouse |
| 40 | Female | 70-79 | High school or vocational college | 5000-10000 | Living with spouse |
| 41 | Female | 70-79 | High school or vocational college | 5000-10000 | Living with spouse |
| 42 | Male | 60-69 | Bachelor’s degree | 10000 and above | Living with spouse |
| 43 | Female | 70-79 | High school or vocational college | 10000 and above | Living with spouse |
| 44 | Female | 70-79 | Master’s degree or above | 10000 and above | Living with spouse |
| 45 | Female | 70-79 | High school or vocational college | 5000-10000 | Living with spouse |
| 46 | Male | 80 and above | High school or vocational college | 5000-10000 | Living with spouse |
| 47 | Male | 70-79 | Bachelor’s degree | 5000-10000 | Living with spouse |
| 48 | Female | 60-69 | Bachelor’s degree | 10000 and above | Living alone |
| 49 | Male | 60-69 | High school or vocational college | 5000-10000 | Living with spouse |
| 50 | Female | 70-79 | High school or vocational college | 5000-10000 | Living with spouse |
| 51 | Female | 70-79 | High school or vocational college | 5000-10000 | Living with spouse |
| 52 | Female | 70-79 | High school or vocational college | 5000-10000 | Living with spouse |
| 53 | Male | 60-69 | High school or vocational college | 5000-10000 | Living with spouse |
| 54 | Male | 70-79 | Bachelor’s degree | 10000 and above | Living with spouse |
| 55 | Female | 70-79 | Bachelor’s degree | 5000-10000 | Living with spouse |
| 56 | Male | 60-69 | High school or vocational college | 5000-10000 | Living with spouse |
| 57 | Female | 70-79 | High school or vocational college | 5000-10000 | Living with children (or spouse and children) |
| 58 | Female | 60-69 | High school or vocational college | 5000-10000 | Living with spouse |
| 59 | Male | 70-79 | High school or vocational college | 5000-10000 | Living with spouse |
| 60 | Female | 70-79 | High school or vocational college | 5000-10000 | Living with spouse |
| 61 | Male | 60-69 | Master’s degree or above | 10000 and above | Living with spouse |
| 62 | Female | 70-79 | High school or vocational college | 5000-10000 | Living with children (or spouse and children) |
| 63 | Male | 70-79 | High school or vocational college | 5000-10000 | Living with children (or spouse and children) |
| 64 | Female | 70-79 | High school or vocational college | 5000-10000 | Living with children (or spouse and children) |
| 65 | Male | 60-69 | Master’s degree or above | 10000 and above | Living with spouse |
| 66 | Male | 60-69 | Master’s degree or above | 10000 and above | Living with spouse |
| 67 | Male | 70-79 | High school or vocational college | 10000 and above | Living with spouse |
| 68 | Female | 70-79 | High school or vocational college | 5000-10000 | Living with spouse |
| 69 | Female | 70-79 | High school or vocational college | 5000-10000 | Living with spouse |
| 70 | Male | 60-69 | High school or vocational college | 10000 and above | Living with spouse |
| 71 | Female | 60-69 | High school or vocational college | 5000-10000 | Living with spouse |
| 72 | Female | 60-69 | Master’s degree or above | 5000-10000 | Living with spouse |
| 73 | Male | 60-69 | High school or vocational college | 10000 and above | Living with spouse |
| 74 | Female | 70-79 | High school or vocational college | 10000 and above | Living with spouse |
| 75 | Female | 60-69 | High school or vocational college | 5000-10000 | Living with spouse |
| 76 | Male | 70-79 | High school or vocational college | 5000-10000 | Living with spouse |
| 77 | Female | 70-79 | High school or vocational college | 5000-10000 | Living with spouse |
| 78 | Male | 70-79 | High school or vocational college | 5000-10000 | Living with spouse |
| 79 | Female | 60-69 | High school or vocational college | 10000 and above | Living with spouse |
| 80 | Female | 70-79 | High school or vocational college | 5000-10000 | Living with spouse |
| 81 | Male | 70-79 | High school or vocational college | 5000-10000 | Living with spouse |
| 82 | Female | 60-69 | High school or vocational college | 5000-10000 | Living with spouse |
| 83 | Female | 70-79 | Junior high school and below | 5000 and below | Living with spouse |
| 84 | Male | 60-69 | Master’s degree or above | 10000 and above | Living with spouse |
| 85 | Male | 70-79 | High school or vocational college | 5000 and below | Living with spouse |
| 86 | Female | 60-69 | High school or vocational college | 5000-10000 | Living with spouse |
| 87 | Female | 70-79 | Junior high school and below | 5000-10000 | Living with spouse |
| 88 | Female | 60-69 | High school or vocational college | 5000-10000 | Living with spouse |
| 89 | Male | 70-79 | High school or vocational college | 5000-10000 | Living with children (or spouse and children) |
| 90 | Male | 70-79 | High school or vocational college | 5000 and below | Living with spouse |
| 91 | Female | 70-79 | High school or vocational college | 5000-10000 | Living with spouse |
| 92 | Male | 60-69 | Bachelor’s degree | 5000-10000 | Living with spouse |
| 93 | Female | 60-69 | Bachelor’s degree | 5000-10000 | Living with spouse |
| 94 | Female | 70-79 | High school or vocational college | 5000-10000 | Living with children (or spouse and children) |
| 95 | Female | 70-79 | High school or vocational college | 10000 and above | Living with spouse |
| 96 | Male | 60-69 | Bachelor’s degree | 5000-10000 | Living with spouse |
| 97 | Male | 70-79 | High school or vocational college | 5000-10000 | Living with spouse |
| 98 | Female | 70-79 | Bachelor’s degree | 10000 and above | Living with spouse |
| 99 | Female | 60-69 | High school or vocational college | 10000 and above | Living with spouse |
| 100 | Female | 60-69 | High school or vocational college | 10000 and above | Living with spouse |

**Table S4.** Basic Information of Survey Respondents in Nanguan Park.

| **Numbe** | **Gender** | **Age** | **Education level** | **Income (RMB/Month)** | **Living status** |
| --- | --- | --- | --- | --- | --- |
| 1 | Female | 60-69 | High school or vocational college | 5000-10000 | Living with spouse |
| 2 | Female | 60-69 | High school or vocational college | 5000-10000 | Living with spouse |
| 3 | Male | 60-69 | Junior high school and below | 5000-10000 | Living with spouse |
| 4 | Female | 70-79 | High school or vocational college | 5000 and below | Living with spouse |
| 5 | Female | 60-69 | Bachelor’s degree | 5000-10000 | Living with spouse |
| 6 | Female | 70-79 | Junior high school and below | 5000-10000 | Living alone |
| 7 | Female | 70-79 | High school or vocational college | 5000-10000 | Living with spouse |
| 8 | Female | 60-69 | High school or vocational college | 5000 and below | Living with spouse |
| 9 | Female | 60-69 | Bachelor’s degree | 5000 and below | Living with children (or spouse and children) |
| 10 | Male | 80 and above | Bachelor’s degree | 5000-10000 | Living with spouse |
| 11 | Male | 60-69 | High school or vocational college | 5000 and below | Living with spouse |
| 12 | Female | 80 and above | Junior high school and below | 5000-10000 | Living with children (or spouse and children) |
| 13 | Male | 60-69 | High school or vocational college | 10000 and above | Living alone |
| 14 | Female | 60-69 | High school or vocational college | 10000 and above | Living with spouse |
| 15 | Female | 60-69 | High school or vocational college | 5000-10000 | Living with spouse |
| 16 | Female | 60-69 | Junior high school and below | 5000-10000 | Living with spouse |
| 17 | Female | 70-79 | Junior high school and below | 5000-10000 | Living with children (or spouse and children) |
| 18 | Female | 70-79 | High school or vocational college | 10000 and above | Living with spouse |
| 19 | Female | 70-79 | Bachelor’s degree | 5000-10000 | Living alone |
| 20 | Female | 60-69 | Bachelor’s degree | 5000-10000 | Living with spouse |
| 21 | Female | 60-69 | High school or vocational college | 5000-10000 | Living with spouse |
| 22 | Male | 60-69 | High school or vocational college | 5000 and below | Living with spouse |
| 23 | Male | 70-79 | Junior high school and below | 5000-10000 | Living with spouse |
| 24 | Female | 60-69 | High school or vocational college | 10000 and above | Living alone |
| 25 | Male | 60-69 | High school or vocational college | 10000 and above | Living with spouse |
| 26 | Male | 60-69 | Junior high school and below | 5000-10000 | Living with children (or spouse and children) |
| 27 | Male | 70-79 | High school or vocational college | 5000-10000 | Living with children (or spouse and children) |
| 28 | Female | 60-69 | High school or vocational college | 5000 and below | Living with spouse |
| 29 | Male | 70-79 | High school or vocational college | 5000-10000 | Living alone |
| 30 | Male | 60-69 | High school or vocational college | 5000-10000 | Living with spouse |
| 31 | Female | 60-69 | Bachelor’s degree | 5000-10000 | Living with spouse |
| 32 | Female | 60-69 | Bachelor’s degree | 10000 and above | Living with spouse |
| 33 | Female | 70-79 | High school or vocational college | 5000 and below | Living with children (or spouse and children) |
| 34 | Female | 70-79 | High school or vocational college | 5000-10000 | Living with children (or spouse and children) |
| 35 | Female | 70-79 | High school or vocational college | 5000 and below | Living with spouse |
| 36 | Male | 70-79 | Junior high school and below | 5000-10000 | Living with spouse |
| 37 | Female | 60-69 | High school or vocational college | 5000-10000 | Living with spouse |
| 38 | Female | 60-69 | High school or vocational college | 5000-10000 | Living with spouse |
| 39 | Male | 60-69 | Master’s degree or above | 10000 and above | Living with children (or spouse and children) |
| 40 | Male | 60-69 | High school or vocational college | 5000-10000 | Living with spouse |
| 41 | Female | 80 and above | Junior high school and below | 5000-10000 | Living with spouse |
| 42 | Female | 60-69 | High school or vocational college | 5000-10000 | Living with spouse |
| 43 | Female | 60-69 | High school or vocational college | 5000-10000 | Living with spouse |
| 44 | Male | 70-79 | Junior high school and below | 10000 and above | Living with spouse |
| 45 | Male | 70-79 | High school or vocational college | 5000-10000 | Living with children (or spouse and children) |
| 46 | Female | 60-69 | High school or vocational college | 10000 and above | Living with spouse |
| 47 | Female | 60-69 | Master’s degree or above | 10000 and above | Living with spouse |
| 48 | Male | 60-69 | Bachelor’s degree | 5000-10000 | Living with spouse |
| 49 | Male | 70-79 | Bachelor’s degree | 10000 and above | Living alone |
| 50 | Female | 70-79 | High school or vocational college | 5000-10000 | Living with spouse |
| 51 | Male | 60-69 | High school or vocational college | 10000 and above | Living with spouse |
| 52 | Male | 60-69 | Junior high school and below | 5000-10000 | Living with spouse |
| 53 | Female | 60-69 | Bachelor’s degree | 10000 and above | Living with spouse |
| 54 | Male | 70-79 | Junior high school and below | 5000-10000 | Living with spouse |
| 55 | Female | 70-79 | High school or vocational college | 5000-10000 | Living with children (or spouse and children) |
| 56 | Female | 60-69 | High school or vocational college | 10000 and above | Living with spouse |
| 57 | Female | 60-69 | Junior high school and below | 5000-10000 | Living with spouse |
| 58 | Female | 60-69 | Master’s degree or above | 10000 and above | Living with spouse |
| 59 | Female | 70-79 | Junior high school and below | 5000-10000 | Living with spouse |
| 60 | Female | 70-79 | High school or vocational college | 5000-10000 | Living with spouse |
| 61 | Female | 60-69 | Bachelor’s degree | 10000 and above | Living with spouse |
| 62 | Male | 60-69 | High school or vocational college | 10000 and above | Living with spouse |
| 63 | Male | 60-69 | Junior high school and below | 5000-10000 | Living with spouse |
| 64 | Female | 80 and above | High school or vocational college | 10000 and above | Living with spouse |
| 65 | Female | 60-69 | Junior high school and below | 5000-10000 | Living with spouse |
| 66 | Female | 60-69 | High school or vocational college | 10000 and above | Living with spouse |
| 67 | Female | 60-69 | High school or vocational college | 10000 and above | Living with spouse |
| 68 | Female | 70-79 | Master’s degree or above | 10000 and above | Living with spouse |
| 69 | Male | 70-79 | Junior high school and below | 5000-10000 | Living alone |
| 70 | Female | 70-79 | High school or vocational college | 5000-10000 | Living with spouse |
| 71 | Male | 70-79 | Junior high school and below | 5000-10000 | Living with spouse |
| 72 | Male | 60-69 | High school or vocational college | 10000 and above | Living with spouse |
| 73 | Female | 60-69 | High school or vocational college | 5000-10000 | Living with spouse |
| 74 | Female | 60-69 | Junior high school and below | 5000-10000 | Living with spouse |
| 75 | Female | 70-79 | Bachelor’s degree | 10000 and above | Living with spouse |
| 76 | Female | 60-69 | Bachelor’s degree | 5000-10000 | Living with spouse |
| 77 | Female | 60-69 | High school or vocational college | 5000-10000 | Living with spouse |
| 78 | Female | 80 and above | High school or vocational college | 5000-10000 | Living with spouse |
| 79 | Male | 70-79 | High school or vocational college | 5000-10000 | Living with spouse |
| 80 | Female | 70-79 | Bachelor’s degree | 5000-10000 | Living with spouse |
| 81 | Female | 70-79 | High school or vocational college | 5000-10000 | Living with spouse |
| 82 | Female | 60-69 | Junior high school and below | 5000-10000 | Living with spouse |
| 83 | Male | 70-79 | High school or vocational college | 5000-10000 | Living with spouse |
| 84 | Male | 60-69 | High school or vocational college | 5000-10000 | Living with spouse |
| 85 | Female | 60-69 | High school or vocational college | 5000-10000 | Living with spouse |
| 86 | Female | 70-79 | Bachelor’s degree | 5000 and below | Living with spouse |
| 87 | Female | 60-69 | High school or vocational college | 5000-10000 | Living with spouse |
| 88 | Female | 60-69 | Bachelor’s degree | 10000 and above | Living with spouse |
| 89 | Male | 60-69 | Bachelor’s degree | 10000 and above | Living with spouse |
| 90 | Female | 70-79 | High school or vocational college | 5000-10000 | Living with spouse |
| 91 | Female | 60-69 | High school or vocational college | 10000 and above | Living with spouse |
| 92 | Male | 80 and above | Bachelor’s degree | 10000 and above | Living with spouse |
| 93 | Female | 60-69 | High school or vocational college | 5000-10000 | Living with spouse |
| 94 | Female | 60-69 | High school or vocational college | 10000 and above | Living with spouse |
| 95 | Male | 60-69 | High school or vocational college | 10000 and above | Living with spouse |
| 96 | Female | 70-79 | Bachelor’s degree | 10000 and above | Living with spouse |
| 97 | Male | 60-69 | High school or vocational college | 5000-10000 | Living with spouse |
| 98 | Male | 80 and above | Junior high school and below | 5000-10000 | Living with spouse |
| 99 | Female | 60-69 | Junior high school and below | 5000-10000 | Living with spouse |
| 100 | Female | 60-69 | High school or vocational college | 10000 and above | Living with children (or spouse and children) |
| 101 | Male | 70-79 | High school or vocational college | 5000-10000 | Living with spouse |
| 102 | Male | 70-79 | High school or vocational college | 10000 and above | Living with spouse |
| 103 | Female | 70-79 | High school or vocational college | 10000 and above | Living with spouse |
| 104 | Female | 60-69 | High school or vocational college | 5000-10000 | Living with spouse |

**Table S5.** Consistency test of the judgment matrix for first-level indicators (B1–B5).

| **Assessment Expert 1** | | | | | | | | |
| --- | --- | --- | --- | --- | --- | --- | --- | --- |
|  | **B1** | **B2** | **B3** | **B4** | **B5** | **Weight** | **CI** | **CR** |
| **B1** | 1 | 6 | 0.333333 | 0.25 | 0.142857 | 0.0797 | 0.11104 | 0.0991 |
| **B2** | 0.166667 | 1 | 0.2 | 0.166667 | 0.142857 | 0.0324 |  |  |
| **B3** | 3 | 5 | 1 | 0.5 | 0.2 | 0.1466 |  |  |
| **B4** | 4 | 6 | 2 | 1 | 0.333333 | 0.2353 |  |  |
| **B5** | 7 | 7 | 5 | 3 | 1 | 0.5059 |  |  |
| **Assessment Expert 2** | | | | | | | | |
|  | **B1** | **B2** | **B3** | **B4** | **B5** | **Weight** | **CI** | **CR** |
| **B1** | 1 | 4 | 0.5 | 0.25 | 0.142857 | 0.0773 | 0.10729 | 0.0958 |
| **B2** | 0.25 | 1 | 0.2 | 0.166667 | 0.142857 | 0.0341 |  |  |
| **B3** | 2 | 5 | 1 | 0.2 | 0.166667 | 0.1053 |  |  |
| **B4** | 4 | 6 | 5 | 1 | 0.333333 | 0.2742 |  |  |
| **B5** | 7 | 7 | 6 | 3 | 1 | 0.5091 |  |  |
| **Assessment Expert 3** | | | | | | | | |
|  | **B1** | **B2** | **B3** | **B4** | **B5** | **Weight** | **CI** | **CR** |
| **B1** | 1 | 2 | 0.25 | 0.5 | 0.166667 | 0.0695 | 0.09213 | 0.0823 |
| **B2** | 0.5 | 1 | 0.25 | 0.333333 | 0.142857 | 0.0471 |  |  |
| **B3** | 4 | 4 | 1 | 3 | 0.142857 | 0.1929 |  |  |
| **B4** | 2 | 3 | 0.333333 | 1 | 0.166667 | 0.1053 |  |  |
| **B5** | 6 | 7 | 7 | 6 | 1 | 0.5852 |  |  |
| **Assessment Expert 4** | | | | | | | | |
|  | **B1** | **B2** | **B3** | **B4** | **B5** | **Weight** | **CI** | **CR** |
| **B1** | 1 | 0.5 | 2 | 5 | 6 | 0.2976 | 0.08545 | 0.0763 |
| **B2** | 2 | 1 | 3 | 4 | 6 | 0.4073 |  |  |
| **B3** | 0.5 | 0.333333 | 1 | 2 | 3 | 0.1507 |  |  |
| **B4** | 0.2 | 0.25 | 0.5 | 1 | 6 | 0.1031 |  |  |
| **B5** | 0.166667 | 0.166667 | 0.333333 | 0.166667 | 1 | 0.0413 |  |  |
| **Assessment Expert 5** | | | | | | | | |
|  | **B1** | **B2** | **B3** | **B4** | **B5** | **Weight** | **CI** | **CR** |
| **B1** | 1 | 0.333333333 | 2 | 3 | 4 | 0.2275 | 0.05505 | 0.0491 |
| **B2** | 3 | 1 | 3 | 4 | 7 | 0.4535 |  |  |
| **B3** | 0.5 | 0.333333333 | 1 | 4 | 5 | 0.1909 |  |  |
| **B4** | 0.333333333 | 0.25 | 0.25 | 1 | 2 | 0.0795 |  |  |
| **B5** | 0.25 | 0.142857143 | 0.2 | 0.5 | 1 | 0.0486 |  |  |
| **Assessment Expert 6** | | | | | | | | |
|  | **B1** | **B2** | **B3** | **B4** | **B5** | **Weight** | **CI** | **CR** |
| **B1** | 1 | 0.5 | 3 | 5 | 7 | 0.3069 | 0.0491 | 0.0438 |
| **B2** | 2 | 1 | 4 | 6 | 7 | 0.4449 |  |  |
| **B3** | 0.333333 | 0.25 | 1 | 3 | 4 | 0.139 |  |  |
| **B4** | 0.2 | 0.166667 | 0.333333 | 1 | 3 | 0.0704 |  |  |
| **B5** | 0.142857 | 0.142857 | 0.25 | 0.333333 | 1 | 0.0388 |  |  |
| **Assessment Expert 7** | | | | | | | | |
|  | **B1** | **B2** | **B3** | **B4** | **B5** | **Weight** | **CI** | **CR** |
| **B1** | 1 | 0.2 | 2 | 3 | 6 | 0.2149 | 0.08572 | 0.0765 |
| **B2** | 5 | 1 | 3 | 5 | 6 | 0.4914 |  |  |
| **B3** | 0.5 | 0.333333 | 1 | 3 | 4 | 0.1664 |  |  |
| **B4** | 0.333333 | 0.2 | 0.333333 | 1 | 3 | 0.0843 |  |  |
| **B5** | 0.166667 | 0.166667 | 0.25 | 0.333333 | 1 | 0.043 |  |  |
| **Assessment Expert 8** | | | | | | | | |
|  | **B1** | **B2** | **B3** | **B4** | **B5** | **Weight** | **CI** | **CR** |
| **B1** | 1 | 0.333333 | 2 | 3 | 5 | 0.2408 | 0.03213 | 0.0287 |
| **B2** | 3 | 1 | 3 | 4 | 7 | 0.459 |  |  |
| **B3** | 0.5 | 0.333333 | 1 | 2 | 3 | 0.1519 |  |  |
| **B4** | 0.333333 | 0.25 | 0.5 | 1 | 3 | 0.1002 |  |  |
| **B5** | 0.2 | 0.142857 | 0.333333 | 0.333333 | 1 | 0.0481 |  |  |
| **Comprehensive Matrix** | | | | | | | | |
|  | **B1** | **B2** | **B3** | **B4** | **B5** | **Weight** | **CI** | **CR** |
| **B1** | 1 | 0.8477 | 1.0905 | 1.4639 | 1.4265 | 0.2244 | 0.00558 | 0.005 |
| **B2** | 1.1797 | 1 | 1.1583 | 1.433 | 1.5651 | 0.2461 |  |  |
| **B3** | 0.917 | 0.8633 | 1 | 1.6012 | 1.1665 | 0.2127 |  |  |
| **B4** | 0.6831 | 0.6979 | 0.6245 | 1 | 1.251 | 0.1615 |  |  |
| **B5** | 0.701 | 0.6389 | 0.8573 | 0.7993 | 1 | 0.1553 |  |  |

**Table S6.** Consistency test of the judgment matrix for second-level indicators (B11–B14).

| **Assessment Expert 1** | | | | | | | |
| --- | --- | --- | --- | --- | --- | --- | --- |
|  | **B11** | **B12** | **B13** | **B14** | **Weight** | **CI** | **CR** |
| **B11** | 1 | 7 | 5 | 4 | 0.5833 | 0.06543 | 0.0735 |
| **B12** | 0.142857 | 1 | 0.5 | 0.166667 | 0.056 |  |  |
| **B13** | 0.2 | 2 | 1 | 0.25 | 0.0954 |  |  |
| **B14** | 0.25 | 6 | 4 | 1 | 0.2654 |  |  |
| **Assessment Expert 2** | | | | | | | |
|  | **B11** | **B12** | **B13** | **B14** | **Weight** | **CI** | **CR** |
| **B11** | 1 | 4 | 5 | 3 | 0.5206 | 0.05335 | 0.0599 |
| **B12** | 0.25 | 1 | 2 | 0.333333 | 0.1195 |  |  |
| **B13** | 0.2 | 0.5 | 1 | 0.166667 | 0.0672 |  |  |
| **B14** | 0.333333 | 3 | 6 | 1 | 0.2927 |  |  |
| **Assessment Expert 3** | | | | | | | |
|  | **B11** | **B12** | **B13** | **B14** | **Weight** | **CI** | **CR** |
| **B11** | 1 | 4 | 7 | 2 | 0.5048 | 0.08467 | 0.0951 |
| **B12** | 0.25 | 1 | 6 | 0.333333 | 0.1552 |  |  |
| **B13** | 0.142857 | 0.166667 | 1 | 0.25 | 0.0513 |  |  |
| **B14** | 0.5 | 3 | 4 | 1 | 0.2888 |  |  |
| **Assessment Expert 4** | | | | | | | |
|  | **B11** | **B12** | **B13** | **B14** | **Weight** | **CI** | **CR** |
| **B11** | 1 | 0.2 | 0.25 | 0.333333 | 0.0695 | 0.0346 | 0.0389 |
| **B12** | 5 | 1 | 2 | 5 | 0.5141 |  |  |
| **B13** | 4 | 0.5 | 1 | 2 | 0.2734 |  |  |
| **B14** | 3 | 0.2 | 0.5 | 1 | 0.1431 |  |  |
| **Assessment Expert 5** | | | | | | | |
|  | **B11** | **B12** | **B13** | **B14** | **Weight** | **CI** | **CR** |
| **B11** | 1 | 0.166667 | 0.25 | 0.5 | 0.0665 | 0.0849 | 0.0954 |
| **B12** | 6 | 1 | 4 | 5 | 0.5789 |  |  |
| **B13** | 4 | 0.25 | 1 | 5 | 0.2616 |  |  |
| **B14** | 2 | 0.2 | 0.2 | 1 | 0.093 |  |  |
| **Assessment Expert 6** | | | | | | | |
|  | **B11** | **B12** | **B13** | **B14** | **Weight** | **CI** | **CR** |
| **B11** | 1 | 0.166666667 | 0.25 | 0.5 | 0.0728 | 0.01033 | 0.0116 |
| **B12** | 6 | 1 | 2 | 4 | 0.5046 |  |  |
| **B13** | 4 | 0.5 | 1 | 3 | 0.3 |  |  |
| **B14** | 2 | 0.25 | 0.333333333 | 1 | 0.1225 |  |  |
| **Assessment Expert 7** | | | | | | | |
|  | **B11** | **B12** | **B13** | **B14** | **Weight** | **CI** | **CR** |
| **B11** | 1 | 0.142857 | 0.166667 | 0.5 | 0.0549 | 0.0602 | 0.0676 |
| **B12** | 7 | 1 | 5 | 6 | 0.6323 |  |  |
| **B13** | 6 | 0.2 | 1 | 2 | 0.2067 |  |  |
| **B14** | 2 | 0.166667 | 0.5 | 1 | 0.1061 |  |  |
| **Assessment Expert 8** | | | | | | | |
|  | **B11** | **B12** | **B13** | **B14** | **Weight** | **CI** | **CR** |
| **B11** | 1 | 0.142857143 | 0.2 | 0.5 | 0.0651 | 0.0280 | 0.0315 |
| **B12** | 7 | 1 | 2 | 3 | 0.4795 |  |  |
| **B13** | 5 | 0.5 | 1 | 4 | 0.335 |  |  |
| **B14** | 2 | 0.333333333 | 0.25 | 1 | 0.1204 |  |  |
| **Comprehensive Matrix** | | | | | | | |
|  | **B11** | **B12** | **B13** | **B14** | **Weight** | **CI** | **CR** |
| **B11** | 1 | 0.5794 | 0.7413 | 0.917 | 0.1903 | 0.01405 | 0.0158 |
| **B12** | 1.726 | 1 | 2.3593 | 1.5501 | 0.3808 |  |  |
| **B13** | 1.3491 | 0.4239 | 1 | 1.1214 | 0.215 |  |  |
| **B14** | 1.0905 | 0.6451 | 0.8918 | 1 | 0.2138 |  |  |

**Table S7.** Consistency test of the judgment matrix for second-level indicators (B21–B24).

| **Assessment Expert 1** | | | | | | | |
| --- | --- | --- | --- | --- | --- | --- | --- |
|  | **B11** | **B12** | **B13** | **B14** | **Weight** | **CI** | **CR** |
| **B11** | 1 | 0.25 | 0.142857 | 0.333333 | 0.0619 | 0.02371 | 0.0266 |
| **B12** | 4 | 1 | 0.5 | 3 | 0.2932 |  |  |
| **B13** | 7 | 2 | 1 | 4 | 0.5125 |  |  |
| **B14** | 3 | 0.333333 | 0.25 | 1 | 0.1325 |  |  |
| **Assessment Expert 2** | | | | | | | |
|  | **B11** | **B12** | **B13** | **B14** | **Weight** | **CI** | **CR** |
| **B11** | 1 | 0.2 | 0.166667 | 0.333333 | 0.0614 | 0.05405 | 0.0607 |
| **B12** | 5 | 1 | 0.333333 | 3 | 0.2826 |  |  |
| **B13** | 6 | 3 | 1 | 3 | 0.5124 |  |  |
| **B14** | 3 | 0.333333 | 0.333333 | 1 | 0.1436 |  |  |
| **Assessment Expert 3** | | | | | | | |
|  | **B11** | **B12** | **B13** | **B14** | **Weight** | **CI** | **CR** |
| **B11** | 1 | 0.25 | 0.2 | 0.333333 | 0.0684 | 0.04472 | 0.0502 |
| **B12** | 4 | 1 | 0.5 | 3 | 0.298 |  |  |
| **B13** | 5 | 2 | 1 | 5 | 0.5063 |  |  |
| **B14** | 3 | 0.333333 | 0.2 | 1 | 0.1273 |  |  |
| **Assessment Expert 4** | | | | | | | |
|  | **B11** | **B12** | **B13** | **B14** | **Weight** | **CI** | **CR** |
| **B11** | 1 | 0.166667 | 0.2 | 0.5 | 0.071 | 0.00822 | 0.0092 |
| **B12** | 6 | 1 | 2 | 3 | 0.4837 |  |  |
| **B13** | 5 | 0.5 | 1 | 2 | 0.2953 |  |  |
| **B14** | 2 | 0.333333 | 0.5 | 1 | 0.15 |  |  |
| **Assessment Expert 5** | | | | | | | |
|  | **B11** | **B12** | **B13** | **B14** | **Weight** | **CI** | **CR** |
| **B11** | 1 | 6 | 7 | 5 | 0.6219 | 0.08561 | 0.0962 |
| **B12** | 0.166667 | 1 | 3 | 0.333333 | 0.1044 |  |  |
| **B13** | 0.142857 | 0.333333 | 1 | 0.166667 | 0.0488 |  |  |
| **B14** | 0.2 | 3 | 6 | 1 | 0.225 |  |  |
| **Assessment Expert 6** | | | | | | | |
|  | **B11** | **B12** | **B13** | **B14** | **Weight** | **CI** | **CR** |
| **B11** | 1 | 6 | 7 | 2 | 0.5314 | 0.02831 | 0.0318 |
| **B12** | 0.166667 | 1 | 3 | 0.333333 | 0.1122 |  |  |
| **B13** | 0.142857 | 0.333333 | 1 | 0.166667 | 0.0524 |  |  |
| **B14** | 0.5 | 3 | 6 | 1 | 0.304 |  |  |
| **Assessment Expert 7** | | | | | | | |
|  | **B11** | **B12** | **B13** | **B14** | **Weight** | **CI** | **CR** |
| **B11** | 1 | 5 | 7 | 2 | 0.5301 | 0.01321 | 0.0148 |
| **B12** | 0.2 | 1 | 2 | 0.5 | 0.1226 |  |  |
| **B13** | 0.142857 | 0.5 | 1 | 0.166667 | 0.0605 |  |  |
| **B14** | 0.5 | 2 | 6 | 1 | 0.2868 |  |  |
| **Assessment Expert 8** | | | | | | | |
|  | **B11** | **B12** | **B13** | **B14** | **Weight** | **CI** | **CR** |
| **B11** | 1 | 6 | 7 | 3 | 0.587 | 0.01905 | 0.0214 |
| **B12** | 0.166667 | 1 | 2 | 0.333333 | 0.1012 |  |  |
| **B13** | 0.142857 | 0.5 | 1 | 0.25 | 0.064 |  |  |
| **B14** | 0.333333 | 3 | 4 | 1 | 0.2478 |  |  |
| **Comprehensive Matrix** | | | | | | | |
|  | **B11** | **B12** | **B13** | **B14** | **Weight** | **CI** | **CR** |
| **B11** | 1 | 1.1067 | 1.1089 | 1.0133 | 0.2631 | 0.00205 | 0.0023 |
| **B12** | 0.9036 | 1 | 1.251 | 1.052 | 0.2602 |  |  |
| **B13** | 0.9018 | 0.7993 | 1 | 0.7813 | 0.2159 |  |  |
| **B14** | 0.9869 | 0.9506 | 1.2799 | 1 | 0.2608 |  |  |

**Table S8.** Consistency test of the judgment matrix for first-level indicators (B31–B35).

| **Assessment Expert 1** | | | | | | | | |
| --- | --- | --- | --- | --- | --- | --- | --- | --- |
|  | **B1** | **B2** | **B3** | **B4** | **B5** | **Weight** | **CI** | **CR** |
| **B1** | 1 | 0.25 | 0.333333 | 3 | 2 | 0.1214 | 0.02243 | 0.02 |
| **B2** | 4 | 1 | 2 | 7 | 6 | 0.4464 |  |  |
| **B3** | 3 | 0.5 | 1 | 7 | 6 | 0.3194 |  |  |
| **B4** | 0.333333 | 0.142857 | 0.142857 | 1 | 0.5 | 0.0447 |  |  |
| **B5** | 0.5 | 0.166667 | 0.166667 | 2 | 1 | 0.0681 |  |  |
| **Assessment Expert 2** | | | | | | | | |
|  | **B1** | **B2** | **B3** | **B4** | **B5** | **Weight** | **CI** | **CR** |
| **B1** | 1 | 0.5 | 0.2 | 2 | 3 | 0.1259 | 0.07398 | 0.0661 |
| **B2** | 2 | 1 | 0.25 | 6 | 7 | 0.2563 |  |  |
| **B3** | 5 | 4 | 1 | 5 | 6 | 0.5011 |  |  |
| **B4** | 0.5 | 0.166667 | 0.2 | 1 | 2 | 0.0706 |  |  |
| **B5** | 0.333333 | 0.142857 | 0.166667 | 0.5 | 1 | 0.0461 |  |  |
| **Assessment Expert 3** | | | | | | | | |
|  | **B1** | **B2** | **B3** | **B4** | **B5** | **Weight** | **CI** | **CR** |
| **B1** | 1 | 0.5 | 0.2 | 0.333333 | 3 | 0.0873 | 0.10446 | 0.0933 |
| **B2** | 2 | 1 | 0.142857 | 0.2 | 6 | 0.1117 |  |  |
| **B3** | 5 | 7 | 1 | 2 | 7 | 0.4774 |  |  |
| **B4** | 3 | 5 | 0.5 | 1 | 5 | 0.2855 |  |  |
| **B5** | 0.333333 | 0.166667 | 0.142857 | 0.2 | 1 | 0.0381 |  |  |
| **Assessment Expert 4** | | | | | | | | |
|  | **B1** | **B2** | **B3** | **B4** | **B5** | **Weight** | **CI** | **CR** |
| **B1** | 1 | 2 | 3 | 0.25 | 0.333333 | 0.1215 | 0.05565 | 0.0497 |
| **B2** | 0.5 | 1 | 4 | 0.142857 | 0.2 | 0.0787 |  |  |
| **B3** | 0.333333 | 0.25 | 1 | 0.142857 | 0.166667 | 0.0402 |  |  |
| **B4** | 4 | 7 | 7 | 1 | 2 | 0.4607 |  |  |
| **B5** | 3 | 5 | 6 | 0.5 | 1 | 0.2988 |  |  |
| **Assessment Expert 5** | | | | | | | | |
|  | **B1** | **B2** | **B3** | **B4** | **B5** | **Weight** | **CI** | **CR** |
| **B1** | 1 | 4 | 5 | 0.166667 | 0.5 | 0.1465 | 0.1068 | 0.0954 |
| **B2** | 0.25 | 1 | 4 | 0.142857 | 0.2 | 0.0649 |  |  |
| **B3** | 0.2 | 0.25 | 1 | 0.142857 | 0.166667 | 0.0344 |  |  |
| **B4** | 6 | 7 | 7 | 1 | 3 | 0.5134 |  |  |
| **B5** | 2 | 5 | 6 | 0.333333 | 1 | 0.2408 |  |  |
| **Assessment Expert 6** | | | | | | | | |
|  | **B1** | **B2** | **B3** | **B4** | **B5** | **Weight** | **CI** | **CR** |
| **B1** | 1 | 2 | 5 | 0.2 | 0.5 | 0.1381 | 0.10575 | 0.0944 |
| **B2** | 0.5 | 1 | 7 | 0.2 | 0.333333 | 0.1033 |  |  |
| **B3** | 0.2 | 0.142857 | 1 | 0.142857 | 0.166667 | 0.0321 |  |  |
| **B4** | 5 | 5 | 7 | 1 | 4 | 0.5121 |  |  |
| **B5** | 2 | 3 | 6 | 0.25 | 1 | 0.2144 |  |  |
| **Assessment Expert 7** | | | | | | | | |
|  | **B1** | **B2** | **B3** | **B4** | **B5** | **Weight** | **CI** | **CR** |
| **B1** | 1 | 3 | 4 | 0.2 | 0.333333 | 0.1329 | 0.05573 | 0.0498 |
| **B2** | 0.333333 | 1 | 2 | 0.166667 | 0.333333 | 0.0719 |  |  |
| **B3** | 0.25 | 0.5 | 1 | 0.142857 | 0.166667 | 0.0434 |  |  |
| **B4** | 5 | 6 | 7 | 1 | 3 | 0.5042 |  |  |
| **B5** | 3 | 3 | 6 | 0.333333 | 1 | 0.2476 |  |  |
| **Assessment Expert 8** | | | | | | | | |
|  | **B1** | **B2** | **B3** | **B4** | **B5** | **Weight** | **CI** | **CR** |
| **B1** | 1 | 2 | 3 | 0.25 | 0.5 | 0.133 | 0.08926 | 0.0797 |
| **B2** | 0.5 | 1 | 6 | 0.142857 | 0.2 | 0.0862 |  |  |
| **B3** | 0.333333 | 0.166667 | 1 | 0.142857 | 0.166667 | 0.0374 |  |  |
| **B4** | 4 | 7 | 7 | 1 | 2 | 0.4651 |  |  |
| **B5** | 2 | 5 | 6 | 0.5 | 1 | 0.2782 |  |  |
| **Comprehensive Matrix** | | | | | | | | |
|  | **B1** | **B2** | **B3** | **B4** | **B5** | **Weight** | **CI** | **CR** |
| **B1** | 1 | 1.251 | 1.3643 | 0.4122 | 0.8409 | 0.1681 | 0.01659 | 0.0148 |
| **B2** | 0.7993 | 1 | 1.7692 | 0.4111 | 0.8294 | 0.1613 |  |  |
| **B3** | 0.733 | 0.5652 | 1 | 0.504 | 0.6514 | 0.1253 |  |  |
| **B4** | 2.426 | 2.4323 | 1.984 | 1 | 2.276 | 0.3599 |  |  |
| **B5** | 1.1892 | 1.2056 | 1.5352 | 0.4394 | 1 | 0.1854 |  |  |

**Table S9.** Consistency test of the judgment matrix for second-level indicators (B41–B44).

| **Assessment Expert 1** | | | | | | | |
| --- | --- | --- | --- | --- | --- | --- | --- |
|  | **B11** | **B12** | **B13** | **B14** | **Weight** | **CI** | **CR** |
| **B11** | 1 | 0.166667 | 0.142857 | 0.25 | 0.0488 | 0.04455 | 0.0501 |
| **B12** | 6 | 1 | 0.333333 | 2 | 0.2486 |  |  |
| **B13** | 7 | 3 | 1 | 5 | 0.5627 |  |  |
| **B14** | 4 | 0.5 | 0.2 | 1 | 0.1398 |  |  |
| **Assessment Expert 2** | | | | | | | |
|  | **B11** | **B12** | **B13** | **B14** | **Weight** | **CI** | **CR** |
| **B11** | 1 | 3 | 0.5 | 7 | 0.3155 | 0.05752 | 0.0646 |
| **B12** | 0.333333 | 1 | 0.2 | 5 | 0.1332 |  |  |
| **B13** | 2 | 5 | 1 | 7 | 0.507 |  |  |
| **B14** | 0.142857 | 0.2 | 0.142857 | 1 | 0.0443 |  |  |
| **Assessment Expert 3** | | | | | | | |
|  | **B11** | **B12** | **B13** | **B14** | **Weight** | **CI** | **CR** |
| **B11** | 1 | 2 | 7 | 3 | 0.4901 | 0.00639 | 0.0072 |
| **B12** | 0.5 | 1 | 5 | 2 | 0.2879 |  |  |
| **B13** | 0.142857 | 0.2 | 1 | 0.333333 | 0.0601 |  |  |
| **B14** | 0.333333 | 0.5 | 3 | 1 | 0.1619 |  |  |
| **Assessment Expert 4** | | | | | | | |
|  | **B11** | **B12** | **B13** | **B14** | **Weight** | **CI** | **CR** |
| **B11** | 1 | 2 | 7 | 4 | 0.5272 | 0.01664 | 0.0187 |
| **B12** | 0.5 | 1 | 3 | 2 | 0.2536 |  |  |
| **B13** | 0.142857 | 0.333333 | 1 | 0.333333 | 0.0684 |  |  |
| **B14** | 0.25 | 0.5 | 3 | 1 | 0.1508 |  |  |
| **Assessment Expert 5** | | | | | | | |
|  | **B11** | **B12** | **B13** | **B14** | **Weight** | **CI** | **CR** |
| **B11** | 1 | 2 | 7 | 4 | 0.49 | 0.02089 | 0.0235 |
| **B12** | 0.5 | 1 | 7 | 4 | 0.3465 |  |  |
| **B13** | 0.142857 | 0.142857 | 1 | 0.5 | 0.0569 |  |  |
| **B14** | 0.25 | 0.25 | 2 | 1 | 0.1065 |  |  |
| **Assessment Expert 6** | | | | | | | |
|  | **B11** | **B12** | **B13** | **B14** | **Weight** | **CI** | **CR** |
| **B11** | 1 | 2 | 7 | 5 | 0.5137 | 0.05938 | 0.0667 |
| **B12** | 0.5 | 1 | 5 | 4 | 0.3158 |  |  |
| **B13** | 0.142857 | 0.2 | 1 | 0.25 | 0.0516 |  |  |
| **B14** | 0.2 | 0.25 | 4 | 1 | 0.1188 |  |  |
| **Assessment Expert 7** | | | | | | | |
|  | **B11** | **B12** | **B13** | **B14** | **Weight** | **CI** | **CR** |
| **B11** | 1 | 2 | 5 | 3 | 0.4658 | 0.02197 | 0.0247 |
| **B12** | 0.5 | 1 | 6 | 2 | 0.3115 |  |  |
| **B13** | 0.2 | 0.166667 | 1 | 0.5 | 0.0715 |  |  |
| **B14** | 0.333333 | 0.5 | 2 | 1 | 0.1512 |  |  |
| **Assessment Expert 8** | | | | | | | |
|  | **B11** | **B12** | **B13** | **B14** | **Weight** | **CI** | **CR** |
| **B11** | 1 | 2 | 7 | 3 | 0.4598 | 0.06621 | 0.0744 |
| **B12** | 0.5 | 1 | 7 | 4 | 0.3494 |  |  |
| **B13** | 0.142857 | 0.142857 | 1 | 0.2 | 0.0457 |  |  |
| **B14** | 0.333333 | 0.25 | 5 | 1 | 0.1451 |  |  |
| **Comprehensive Matrix** | | | | | | | |
|  | **B11** | **B12** | **B13** | **B14** | **Weight** | **CI** | **CR** |
| **B11** | 1 | 1.5422 | 2.9668 | 2.8002 | 0.4143 | 0.01413 | 0.0159 |
| **B12** | 0.6484 | 1 | 2.4884 | 2.9084 | 0.3223 |  |  |
| **B13** | 0.3371 | 0.4019 | 1 | 0.6852 | 0.1209 |  |  |
| **B14** | 0.3571 | 0.3438 | 1.4593 | 1 | 0.1425 |  |  |

**Table S10.** Consistency test of the judgment matrix for second-level indicators (B51–B54).

| **Assessment Expert 1** | | | | | | | |
| --- | --- | --- | --- | --- | --- | --- | --- |
|  | **B11** | **B12** | **B13** | **B14** | **Weight** | **CI** | **CR** |
| **B11** | 1 | 0.2 | 0.5 | 0.333333 | 0.0815 | 0.02689 | 0.0302 |
| **B12** | 5 | 1 | 6 | 2 | 0.5307 |  |  |
| **B13** | 2 | 0.166667 | 1 | 0.333333 | 0.1101 |  |  |
| **B14** | 3 | 0.5 | 3 | 1 | 0.2777 |  |  |
| **Assessment Expert 2** | | | | | | | |
|  | **B11** | **B12** | **B13** | **B14** | **Weight** | **CI** | **CR** |
| **B11** | 1 | 0.333333 | 6 | 0.5 | 0.1865 | 0.03674 | 0.0413 |
| **B12** | 3 | 1 | 7 | 2 | 0.4748 |  |  |
| **B13** | 0.166667 | 0.142857 | 1 | 0.166667 | 0.0468 |  |  |
| **B14** | 2 | 0.5 | 6 | 1 | 0.2919 |  |  |
| **Assessment Expert 3** | | | | | | | |
|  | **B11** | **B12** | **B13** | **B14** | **Weight** | **CI** | **CR** |
| **B11** | 1 | 2 | 5 | 4 | 0.4693 | 0.05264 | 0.0591 |
| **B12** | 0.5 | 1 | 6 | 4 | 0.3473 |  |  |
| **B13** | 0.2 | 0.166667 | 1 | 0.333333 | 0.0606 |  |  |
| **B14** | 0.25 | 0.25 | 3 | 1 | 0.1228 |  |  |
| **Assessment Expert 4** | | | | | | | |
|  | **B11** | **B12** | **B13** | **B14** | **Weight** | **CI** | **CR** |
| **B11** | 1 | 7 | 3 | 6 | 0.5779 | 0.04177 | 0.0469 |
| **B12** | 0.142857 | 1 | 0.2 | 0.333333 | 0.0539 |  |  |
| **B13** | 0.333333 | 5 | 1 | 3 | 0.2579 |  |  |
| **B14** | 0.166667 | 3 | 0.333333 | 1 | 0.1102 |  |  |
| **Assessment Expert 5** | | | | | | | |
|  | **B11** | **B12** | **B13** | **B14** | **Weight** | **CI** | **CR** |
| **B11** | 1 | 7 | 4 | 6 | 0.6018 | 0.08497 | 0.0955 |
| **B12** | 0.142857 | 1 | 0.166667 | 0.2 | 0.0439 |  |  |
| **B13** | 0.25 | 6 | 1 | 2 | 0.22 |  |  |
| **B14** | 0.166667 | 5 | 0.5 | 1 | 0.1343 |  |  |
| **Assessment Expert 6** | | | | | | | |
|  | **B11** | **B12** | **B13** | **B14** | **Weight** | **CI** | **CR** |
| **B11** | 1 | 7 | 4 | 5 | 0.595 | 0.04281 | 0.0481 |
| **B12** | 0.142857 | 1 | 0.2 | 0.5 | 0.0598 |  |  |
| **B13** | 0.25 | 5 | 1 | 3 | 0.2407 |  |  |
| **B14** | 0.2 | 2 | 0.333333 | 1 | 0.1045 |  |  |
| **Assessment Expert 7** | | | | | | | |
|  | **B11** | **B12** | **B13** | **B14** | **Weight** | **CI** | **CR** |
| **B11** | 1 | 6 | 2 | 3 | 0.47 | 0.05018 | 0.0564 |
| **B12** | 0.166667 | 1 | 0.25 | 0.5 | 0.0729 |  |  |
| **B13** | 0.5 | 4 | 1 | 5 | 0.3412 |  |  |
| **B14** | 0.333333 | 2 | 0.2 | 1 | 0.1159 |  |  |
| **Assessment Expert 8** | | | | | | | |
|  | **B11** | **B12** | **B13** | **B14** | **Weight** | **CI** | **CR** |
| **B11** | 1 | 7 | 3 | 4 | 0.5525 | 0.02602 | 0.0292 |
| **B12** | 0.142857 | 1 | 0.166667 | 0.5 | 0.0603 |  |  |
| **B13** | 0.333333 | 6 | 1 | 2 | 0.2581 |  |  |
| **B14** | 0.25 | 2 | 0.5 | 1 | 0.1291 |  |  |
| **Comprehensive Matrix** | | | | | | | |
|  | **B11** | **B12** | **B13** | **B14** | **Weight** | **CI** | **CR** |
| **B11** | 1 | 2.573 | 2.8473 | 2.482 | 0.4658 | 0.00674 | 0.0076 |
| **B12** | 0.3887 | 1 | 0.7172 | 0.7774 | 0.1539 |  |  |
| **B13** | 0.3512 | 1.3943 | 1 | 1.1624 | 0.1959 |  |  |
| **B14** | 0.4029 | 1.2864 | 0.8603 | 1 | 0.1843 |  |  |

**Table S11.** Reliability Test Results of the Questionnaire.

| **Name** | **Reliability Testing Method** | **B11: Accessibility** | **B2: Security** | **B3: Comfort** | **B4: Functional Diversity** | **B5: Social Supportive** | **Community Park Age-Friendly Evaluation A** |
| --- | --- | --- | --- | --- | --- | --- | --- |
| Wanshou Park | Cronbach’s α | 0.722 | 0.695 | 0.701 | 0.756 | 0.714 | 0.736 |
| Shuangxiu Park | Cronbach’s α | 0.719 | 0.705 | 0.673 | 0.748 | 0.725 | 0.756 |
| Nanguan Park | Cronbach’s α | 0.731 | 0.706 | 0.713 | 0.806 | 0.716 | 0.773 |

**Table S12. Validity Test Results of the Questionnaire.**

| **Name** | **Reliability Testing Method** | | **B11: Accessibility** | **B2: Security** | **B3: Comfort** | **B4: Functional Diversity** | **B5: Social Supportive** | **Community Park Age-Friendly Evaluation A** |
| --- | --- | --- | --- | --- | --- | --- | --- | --- |
| Wanshou Park | KMO | | 0.713 | 0.736 | 0.720 | 0.753 | 0.753 | 0.645 |
|  | Bartlett’s Test of Sphericity | Approximate Chi-square | 83.918 | 66.176 | 91.805 | 99.286 | 72.527 | 586.658 |
|  |  | Degrees of Freedom | 6 | 6 | 10 | 6 | 6 | 210 |
|  |  | Significance | <0.001 | <0.001 | <0.001 | <0.001 | <0.001 | <0.001 |
| Shuangxiu Park | KMO | | 0.731 | 0.740 | 0.635 | 0.727 | 0.732 | 0.654 |
|  | Bartlett’s Test of Sphericity | Approximate Chi-square | 74.716 | 66.451 | 79.303 | 93.251 | 79.232 | 573.950 |
|  |  | Degrees of Freedom | 6 | 6 | 10 | 6 | 6 | 210 |
|  |  | Significance | <0.001 | <0.001 | <0.001 | <0.001 | <0.001 | <0.001 |
| Nanguan Park | KMO | | 0.739 | 0.739 | 0.734 | 0.790 | 0.739 | 0.707 |
|  | Bartlett’s Test of Sphericity | Approximate Chi-square | 87.713 | 70.877 | 93.604 | 126.457 | 74.476 | 619.175 |
|  |  | Degrees of Freedom | 6 | 6 | 10 | 6 | 6 | 210 |
|  |  | Significance | <0.001 | <0.001 | <0.001 | <0.001 | <0.001 | <0.001 |

**Equation S1. Consistency Test Formula of the Judgment Matrix.**

1）The formula for calculating the consistency index CI is shown in Equation (1).

$\mathrm{CI}=\frac{\lambda_{max}-n}{(n-1)}$ （1）

2）The formula for calculating the consistency ratio CR is shown in Equation (2).

$CR=\frac{CI}{RI}=\frac{\lambda_{max}-n}{(n-1)RI}<0.1$ （2）

3）$\lambda_{max}$To determine the maximum eigenvalue of the matrix, the calculation formula is shown in Equation (6).$\lambda_{max}$ is the largest eigenvalue of the matrix，Among them, $Ā$ is the judgment integration matrix,$W$ is the weight vector,$W_{i}$ represents the i-th component of the weight vector W.

$\lambda_{max}=\sum_{i=1}^{n} \frac{{[ĀW]}_{i}}{{nW}_{i}}$ （3）
